# Cdc48 plays a crucial role in redox homeostasis through dynamic reshaping of its interactome during early stationary phase

**DOI:** 10.1016/j.redox.2025.103651

**Published:** 2025-05-01

**Authors:** Meytal Radzinski, Tal Oppenheim, Ohad Yogev, Adi Levy, Melamed-Book Naomi, Assaf Kacen, Yifat Merbl, Tommer Ravid, Dana Reichmann

**Affiliations:** a Department of Biological Chemistry, The Alexander Silberman Institute of Life Sciences, Safra Campus Givat Ram, The Hebrew University of Jerusalem, Jerusalem, 9190401, Israel; b Bio-Imaging Unit, The Alexander Silberman Institute of Life Sciences, Safra Campus Givat Ram, The Hebrew University of Jerusalem, Jerusalem, 9190401, Israel; c Department of Immunology, The Weizmann Institute of Science, Rehovot, 7610001, Israel; d The Center for Nanoscience and Nanotechnology, Safra Campus Givat Ram, The Hebrew University of Jerusalem, 9190401, Israel

## Abstract

Most microbial cells on earth predominantly exist in non-proliferating, dormant conditions, such as the stationary state. The stationary phase is a crucial stage during the cellular lifespan, which requires homeostatic rewiring for long-term viability and rapid responses to environmental changes. Here, we show that entry to the stationary phase in yeast is accompanied by increased cytosolic and mitochondrial oxidation, imposing stress on the proteostasis network. We establish a functional link between redox and protein homeostasis, mediated by a key protein quality control member, Cdc48/p97/VCP.

Comparative proteomic analysis of post-mitotic yeast cells reveals that while the global proteome remains largely stable during the first stages of stationary phase, the Cdc48 interactome undergoes significant remodeling, including altered interactions with antioxidants and its cofactors Shp1/Ubx1 and Ubx2.

To challenge yeast Cdc48's capacity as a redox-switch protein during the early stages of the stationary phase, we utilized redox proteomics to map changes in reversible oxidation modification on Cdc48's cysteines upon entry to the stationary phase. We revealed the temporal and reversible oxidation of Cdc48-Cys115 as a key regulatory event essential for stationary-phase survival and interactome modulation. Cys115-to-serine mutation significantly reduced longevity and increased oxidative stress sensitivity, correlating with disrupted interactions between Cdc48 and antioxidants, and cofactor Shp1, specifically with the phosphorylated form of Shp1.

Taken together, these findings identify a new thiol switch protein in the protein degradation pathway, while further defining novel roles for Cdc48 in reshaping the proteome during the yeast stationary phase.

## 1 Introduction

The cellular proteome undergoes significant and dynamic changes throughout different stages of cell growth and life. These changes reflect varying metabolic demands, alongside morphological shifts and adaptations to diverse environmental challenges, during both proliferative growth and the stationary (i.e., quiescent) phase [[1], [2], [3]]. Cells in the stationary phase in particular are exposed to a greater degree of cellular and environmental stressors challenging protein homeostasis in bacteria [[4], [5], [6]] and eukaryotes [[7], [8], [9]]. This includes the accumulation of oxidants, changes in pH, and nutrient deficiency at later stages, leading to altered protein biogenesis, differences in post-translational modification (PTM) (e.g., oxidation) profiles, and degradation [4,8,[10], [11], [12], [13], [14], [15], [16], [17], [18]].

Entry to the stationary phase is tightly regulated and very well characterized [1,17,19,20], with studies showing relocation of proteasomes from the nucleus to the periphery, the formation of stress granules in the cytosol [[21], [22], [23], [24]], and a reshaping of protein quality control (PQC) pathways [[25], [26], [27]]. Most of these studies have focused on glucose or stress-induced prolonged quiescence, which results in substantial metabolic changes, protein misfolding, and the accumulation of stress-specific PTMs (including oxidation) [9]. However, the early stages and onset of these processes remain poorly understood.

Notably, reduced proteasome and chaperone activity during the stationary phase result in the misfolding of oxidized proteins [26,28]. These highlight both the elevated protein oxidation in the stationary phase and the critical role of the protein homeostasis system in restoring the redox balance of the cellular proteome in the postmitotic phase. This is not surprising, as proteins are one of the primary targets of cellular oxidants due to high abundance of oxidation-sensitive amino acids and the potential to form non-native covalent bonds, which can disrupt their structure and function. While protein oxidation often leads to a protein loss-of-function, subsequent misfolding and degradation, site-specific modification of a distinct class of redox-regulatory proteins can facilitate a gain-of-function. Such regulatory proteins, named thiol switches, are activated or deactivated by specific, typically reversible, thiol modification (e.g., Sulfenylation (S–OH), Glutathionylation, disulfide bond formation, or others) in response to changes in the redox status of cells [[29], [30], [31]]. Thiol switch proteins are a fundamental component of the regulatory mechanisms that govern redox homeostasis, from prokaryotes all the way through humans. Their reactive thiols usually have unique chemical properties, while some of the thiols themselves are located in structurally flexible and conserved regions [[32], [33], [34], [35], [36]].

Thiol switch proteins are diverse in their function and structures, ranging from well-studied antioxidant enzymes, transcriptional factors, and molecular chaperones [13,29,[37], [38], [39], [40], [41], [42], [43]]. In the context of the current study, some of these redox regulators were shown to be crucial for stationary phase viability, such as thioredoxins, Trx1 and Trx2, in yeast [12,44]. Here, we focus on a novel potential redox switch protein, the yeast AAA + ATPase Cdc48 (VCP/p97 in mammals, Ter94 in *Drosophila*), one of the key players in protein quality control (PQC) [[45], [46], [47], [48], [49], [50]].

This study characterizes very early events during the stationary phase in yeast cells, dissecting the transition from the onset to initial stages of the stationary phase, focusing on the interplay between redox and protein homeostasis. We show that during this transition, intracellular oxidation doubles, in line with oxidation of the Cdc48 thiol group at Cys 115.

Cdc48 is primarily known for its role in assisting distinct degradation pathways in the cell, particularly ER-associated degradation (ERAD) [[51], [52], [53], [54], [55]], mitochondria-associated degradation (MAD) [[56], [57], [58]], chromatin-associated degradation (CAD) [59], and more [60,61]. Promiscuous and highly abundant, Cdc48 interacts with many cofactors, including the canonical Npl4-Ufd1 complex, the UBX-family of proteins (such as Shp1/Ubx1, Ubx2), Vms1, and others [[62], [63], [64]], each of which mediates specific PQC-related processes across different cellular compartments. These cofactors serve as adaptors, facilitating the recruitment of the misfolded proteins and mediating their interaction with Cdc48. To fulfill its function, Cdc48/VCP comprises a short C-terminal domain, two ATPase domains necessary for substrate transfer and partial unfolding, and the N-terminal domain, which is essential for binding UBX-family cofactors, Ufd1, and Npl4. Thus, the N-terminal domain serves as the gears while the ATPase domains are the engine of Cdc48 [49,62,[64], [65], [66], [67], [68]].

Here, we show that oxidation of Cdc48-Cys115, located in the N-terminal domain of Cdc48, is necessary for healthy growth and survival during the early stationary phase in yeast and coping with oxidative stress conditions. By conducting an extensive proteomic analysis of changes in post-mitotic yeast cells during the early stationary phase, we show that while the proteome itself undergoes only minor perturbations, the interactome of Cdc48 specifically undergoes substantial changes. These changes are defined by increased interactions with redox-associated proteins, mitochondrial factors, and others (including the canonical Cdc48 cofactor Shp1/Ubx1), indicating a major shift in Cdc48's subcellular environment.

We identify Cys115 as the source of some of these changes, finding that it alone among Cdc48's cysteine residues undergoes site-specific oxidation during the early stages of the stationary phase. Using a redox-insensitive Cys-to-Ser mutation, we find that the capacity of Cys115 to undergo reversible oxidation is specifically linked with Cdc48's increased association with cellular antioxidants and with its cofactor Shp1, more specifically with the phosphorylated form of Shp1. We suggest a model by which a novel redox switch cysteine residue in Cdc48 contributes to regulating the cellular oxidative response during the first stages of stationary phase associated with increased intracellular oxidation.

## 2 Methods

### 2.1 Strain preparation

Plasmids containing both WT and C115S Cdc48 were prepared C-terminally tagged with FLAG and HIS-tag sequences. Plasmids are based on pRS415(LEU2), as used in Oppenheim et al. [74].

Shp1-GFP strains were prepared through colony PCR of a chromosomally tagged Shp1-GFP strain with HIS selection, kindly provided by Nir Friedman. Primers were targeted to match a sequence upstream the C-terminal end of Shp1 (forward), along with a reverse primer downstream the end of the gene. Inserts were transformed by homologous recombination into FLAG-tagged Cdc48 strains (with and without the C115S mutation) using a protocol modified from Gietz and Schiestl [92] and confirmed by PCR and sequencing.

### 2.2 Growth mediums

Growth was conducted on a partially synthetic, casein-supplemented medium (semi-rich): 0.017 % yeast nitrogen base (w/o amino acids and ammonium sulfate), 0.5 % ammonium sulfate, 2 % glucose (unless otherwise specified), 0.2 % casamino acid mix, 0.000004 % Trp, 0.000005 % Thr (amino acids to excess; subject to minor variation), 4 ml 1 % adenine in 1-L DDW, autoclaved or filtered. The medium may also be supplemented with uracil, depending on strain selection.

### 2.3 Seahorse assay

Sensor plate was incubated with 500 μL calibration buffer overnight at 30 °C, followed by 3 h of sensor soaking for sensor calibration (as described in Radzinski et al. [70]). Approximately 1.5 x 10^6^ cells were loaded onto the ConA treated wells, seeded by centrifugation at 100×*g* for 1 min. After equilibration, six cycles were run, with 3 min of sample mixing, a 2-min wait period, and 3 min of measurement of basal oxygen consumption rates (OCR).

### 2.4 Yeast lysis

For co-immunoprecipitation, samples were grown on 2–4 ml synthetic medium supplemented with casein amino acid, then diluted to 0.2 O.D._600_ on 15 ml medium and grown for varying degrees of time. Cells were harvested and broken in “Extraction buffer” (0.5 ml Tris HCl pH 7.5 buffer with protease inhibitors) (all stages conducted on ice or at 4 °C), using glass beads and 4 cycles of disruption (20 s pulse, 30 s on ice). Cells were then diluted with 260 μl Extraction buffer and 40 μl 20 % Triton in order to solubilize membranes and centrifuged at low speed (0.4×*g*) for 5 min in order to clear broken cells. Supernatant was then centrifuged at 13,300×*g* for 30 min, after which the supernatant was transferred to a new 2 ml tube. Supernatant was diluted 1:1 with “Resuspension buffer” (50 mM Tris-HCl, pH 7.5, 200 mM NaAc, 10 % glycerol) and protease inhibitors. Magnetic beads were prepared in buffer and added to each sample and incubated overnight at 4 °C under constant agitation. Next morning, samples were cleared of the supernatant and beads washed several times before proceeding to sample preparation for mass spectrometry.

For all-proteome analysis, 5 mL of stationary phase yeast cultures (24 h, 48 h) were lysed using 300 μl 0.2 M NaOH and resuspended using a lysis buffer (100 mM DTT [15.425 mg DTT], 100 μl 1 M Tris HCl pH 7.5, 100 μl SDS 20 %, complete with DDW to 1 ml), before being taken to mass spectrometry preparation.

### 2.5 Mass spectrometry preparation

Samples were then diluted using 400 μl of a urea buffer (8 M urea in 0.1 M Tris HCl pH 8.5), loaded onto a filter, and centrifuged for 10 min at 12,000×*g* following the standard FASP protocol [47]. This process was repeated three times, with flow-through discarded, after which samples were incubated in the dark for 60 min with 0.5 M iodoacetamide (IAM) and urea buffer (final iodoacetamide concentration of 0.05 M), under constant agitation (350 RPM, 25 °C). Samples were then washed again three times with urea buffer and twice with digestion buffer (10 % ACN, 25 mM Tris HCl pH 8.5), then centrifuged at 12,000×*g* for 8 min. Filters were transferred to a new collection tube, suspended in 300 μl of digestion buffer with 1 μl of trypsin (Promega), mixed for 1 min at 600 rpm, and left overnight at 350 rmp, 37 °C. Following digestion, samples were centrifuged for 10 min at 12,000 g.

For cysteine-labeling, samples were treated with 0.005 M TCEP following IAM incubation, for 60 min under constant agitation (350 RPM, 25 °C). This was then followed by treatment with N-ethylmaleimide (NEM) in Urea buffer (final NEM concentration of 0.05 M), incubated for 60 min under constant agitation (350 RPM, 25 °C), before continuing with the wash stages as described above.

The peptide concentration was determined, after which the peptides were loaded onto stage tips in equal amounts. Stage tips were activated using 100 μl MS-grade methanol (100 % MeOH) and centrifuged for 2 min at 2000 g, after which they were cleaned with 100 μl of elution buffer (80 % ACN, 0.1 % formic acid) and centrifuged again for 2 min at 2000 g. The stage tips were returned to their hydrophilic state by suspension in 100 μl of buffer A (0.1 % HPLC-grade TFA) and centrifugation at 2000×*g* for 2 min, repeated once. 10–30 μg of protein was then loaded per stage tip (as per protein preparation above), and centrifuged at 1000×*g* for 2 min. Proteins were then washed twice with 100 μl buffer A at 1000×*g* for 2 min and transferred to a new collection tube. Peptides were eluted using 60 μl buffer B (80 % ACN, 0.1 % HPLC-grade TFA) centrifuged at 250×*g* for 2 min, and another 30 μl buffer B centrifuged at 250×*g* for 2 min. Samples were then dried using a SpeedVac for 24 min at 1300 rpm at 35 °C, after which they were dissolved in 6–12 μl of buffer A and prepared for tandem mass spectrometry analysis.

### 2.6 Nano-LC-MS/MS analysis

The peptides were injected into a Nano Trap Column, 100 μm i.d. × 2 cm, packed with Acclaim PepMap100C18, 5 μm, 100 Å (Thermo Scientific) for 8 min at flow 5ul/min, and then separated on a C18 reverse-phase column coupled to the Nano electrospray, EASY-spray (PepMap, 75 mm × 50 cm, Thermo Scientific) at flow 300 nl/min using an Dionex Nano-HPLC system (Thermo Scientific) coupled online to Orbitrap Mass spectrometer, Q Exactive Plus (Thermo Scientific). To separate the peptides, the column was applied with a linear gradient with a flow rate of 300 nl/min at 45 °C: from 1 to 35 % in 100 min, from 35 to 55 % in 43 min, from 55 to 90 % in 5 min, and held at 90 % for an additional 30 min, and then equilibrated at 1 % for 20 min (solvent A is 0.1 % formic acid, and solvent B is 80 % acetonitrile, 0.1 % formic acid). The Q Exactive was operated in a data-dependent mode. The survey scan range was set to 200–2000 *m*/*z*, with a resolution of 70,000 at m/z. Up to the 12 most abundant isotope patterns with a charge of ≥2 and less than 7 were subjected to higher-energy collisional dissociation with a normalized collision energy of 28, an isolation window of 1.5 *m*/*z*, and a resolution of 17,500 at m/z. To limit repeated sequencing, dynamic exclusion of sequenced peptides was set to 60 s. Thresholds for ion injection time and ion target value were set to 70 ms and 3 × 10^6^ for the survey scans and to 70 ms and 10^5^ for the MS/MS scans. Only ions with “peptide preferable” profile were analyzed for MS/MS. Data was acquired using Xcalibur software (Thermo Scientific). Column wash with 80 % ACN for 40 min was carried out between each sample run to avoid potential carryover of the peptides.

### 2.7 Data analysis and statistics of the proteomic data

For protein identification and quantification, we used MaxQuant software [75,93], versions 1.5.3.30 and 1.6.3.3. We used Andromeda search incorporated into MaxQuant to search for MS/MS spectra against the UniProtKB database of Saccharomyces cerevisiae proteome, (Uniprot release, Aug 2016). The identification allowed two missed cleavages. Enzyme specificity was set to trypsin, allowing N-terminal to proline cleavage and up to two miscleavages. Peptides had to have a minimum length of seven amino acids to be considered for identification. Carbamidomethylation was set as a fixed modification, and methionine oxidation was set as a variable modification. A false discovery rate (FDR) of 0.05 was applied at the peptide and protein levels. An initial precursor mass deviation of up to 4.5 ppm and fragment mass deviation up to 20 ppm were allowed. Only proteins identified by more than 2 peptides in at least one sample were considered. To quantify changes in protein expression we used the label-free quantification (LFQ) using the MaxQuant default parameters. For statistical and bioinformatic analysis, we used Perseus software (http://141.61.102.17/perseus_doku/doku.php?id=start). For functional enrichment analysis, the DAVID webserver [89] was used, as well the UniProt database and the Yeast Genome Database [90] for both localization and additional functional enrichment analysis. Protein localization was determined using the DeepLoc server [51].

Identification of cysteine modifications using NEM and IAM was done using Proteome Discoverer (Thermo Fisher Scientific). Proteomic data were uploaded to the PRIDE database with the dataset identifier: PXD045122.

### 2.8 Grx1-roGFP2 probes and cell survival FACS analysis

The strains were transformed with cytosolic or mitochondrial Grx1-roGFP2 (kindly provided by Bruce Morgan). Transformations were carried out using a standard “One Step” protocol, modified from Chen et al. [94]. Cultures were diluted to an OD_600_ of approximately 0.25 to begin growth and grown at 30 °C under constant agitation. Transformations were refreshed every 2–3 weeks in an adapted version of the protocol from Chen et al. [52]. Cell survival was assessed using propidium iodide treatment per protocol modified from Ocampo and Barrientos [95]. Fluorescence was measured by FACS per protocols described in Radzinski et al. [70].

The degree of oxidation (OxD) was calculated based on fluorescence intensity ratios at excitation wavelengths of 405 nm and 488 nm for the Grx1-roGFP2 probe as described in Radzinski et al. [70]. Briefly, cells expressing Grx1-roGFP were excited at 405 nm and 488 nm, and emission was recorded at 510 nm. To determine degree of oxidation, the observed I405/I488 ratios were normalized to those obtained in fully reduced (10 mM DTT) and fully oxidized (8 mM diamide) cells and calculated according to Eq. (1):

$$OxD_{roGFP2}=\frac{I405sample*I488_{red}-I405_{red}*I488sample}{I405sample*I488_{red}-I405sample*I488_{ox}+I405_{ox}*I488sample-I405_{red}I488sample}$$

### 2.9 Monitoring yeast growth using plate reader

Growth curves were conducted on freshly diluted yeast samples using a TECAN Infinite 200 PRO or SPARK series. Treatment with reagents (i.e., H_2_O_2_, DTT) was conducted directly within the wells during growth, with the reagent added to the growth medium into which the yeast cultures were diluted. OD_600_ measurements and minimal doubling times were analyzed using MDTCalc [96].

### 2.10 Sorting using Grx1-roGFP2 probe

750,000 cells were sorted by their oxidative status after ∼64 h of growth according to method described in Radzinski et al. [70] using flow cytometry, centrifuged at 3000×*g* for 5 min, and resuspended in 200 μL selective growth medium. Growth curve was conducted as described above.

### 2.11 NEM-IAM preparation and data analysis

Samples were prepared using co-immunoprecipitation method as previously described (growing only 5 mL of yeast), followed by modified FASP protocol. All buffers for both co-IP and FASP were first placed in an anaerobic chamber to reduce oxidation during sample preparation. Samples were treated with 0.05 M IAM as in the previously described FASP protocol, after which they were washed twice with urea and treated with 0.005 M TCEP. Samples were washed twice more with urea and then treated with 0.05 M NEM. All treatments were carried out for 60 min under 350 rpm shaking at 25 °C. Remaining FASP, stage tips, and mass spectrometry protocols followed as previously described. Peptide identification, quantification, and spectrum visualization was done using Proteome Discoverer (Thermo Fisher Scientific), identifying all peptides containing the cysteine residue of interest. The intensity of each identified peptide with either the IAM or NEM modification (high confidence) was summed and the ratio between them defined the degree of oxidation (Fig. 2B).

### 2.12 Cell imaging

Mitochondrial imaging was conducted using mitochondrially-targeted roGFP2 probes (transformed per previous protocol), with samples grown overnight, diluted, and imaged at the log phase. Images were taken using the FV-1200 confocal microscope (Olympus., Japan) using a 60X/1.42 oil objective. GFP was excited with a 488 nm laser and emission was collected at 500–540 nm, alongside a DIC image. Z-stack images were compiled using ImageJ.

For Shp1-GFP imaging, strains were grown overnight and diluted 1:10. Cells were then imaged at 4.5 (log phase), 24, or 48 h h. Imaging was performed using an automated Olympus SpinSR system using a Hamamatsu flash Orca 4.0 camera and a CSUW1-T2SSR SD Yokogawa spinning disk Unit with a 50 μm pinhole disk. Images were acquired using a 60 × oil lens (NA 1.42) (Olympus), 100 mW 488 nm OBIS LX laser system (Coherent), FITC Filter Quad Emission filter: 515/30 (Chroma).

### 2.13 Post-translational modification (PTM) search through the PROMISE pipeline

We used the PROMISE 1 pipeline to search the MS data generated for the interactome analysis for post-translational modifications [27], using a subgroup FDR of 0.01. Forty-five different PTM combinations were searched. We focused on Oxidation (single Oxidation on WHKPMC, Dioxidation on PRKMYC, and trioxidation on cysteine) as well as other modifications on cysteine, like cysteinylation, palmitoylation, carbamidomethylation, and nitrosylation, together with phosphorylation, methylation, ubiquitination, citrullination, ADP-Ribosyl, acetylation, and deamidation.

### 2.14 Isothermal titration calorimetry assay

ITC measurements were done at 25 °C, using the Malvern MicroCal PEAQ-ITC apparatus. 220 μM Shp1 were injected into 16 μM Cdc48, in either the WT or -C115S variant. The injection volumes were 2 μL, injected for 4 s duration, and 150 s spacing between each injection. The data were gathered and analyzed in Malvern MicroCal PEAQ-ITC software.

### 2.15 Yeast spot assay

Strains were grown overnight and then diluted to 0.2 O.D and grown to the logarithmic phase. 1 O.D. of cells were taken, washed, and serially diluted of 1:10 in DDW before growth on selective plates for 2 days.

### 2.16 Statistical analyses

All statistical tests in this paper were conducted using Excel, unless otherwise stated. Specific statistical tests are referred to in the corresponding figure legend. For all cases of a Student's t-test done in Excel, a two-tailed distribution with two-samples of equal variance was used. Standard p-value cutoffs were used for defining the significance: NS (non-significant) – p-value >0.05, ∗ - p-value <0.05, ∗∗ - p-value <0.01, ∗∗∗ - p-value <0.005.

All data in bar graphs are displayed as mean ± SD unless otherwise noted. Replicate numbers are listed in each figure legend.

## 3 Results

### 3.1 Analysis of the thiol redox state of Cdc48 during early stationary growth

The in-depth characteristics of cellular oxidation and aging have both been addressed across a wide range of organisms and cellular conditions. In this context, both others and we have established the occurrence of increased cellular oxidation during chronological aging of yeast populations [13,14,69,70]. To deepen and extend this effort, we have currently defined the redox status, proteomic profile, and Cdc48 interactome during early stages of stationary growth.

**Fig. 1:**
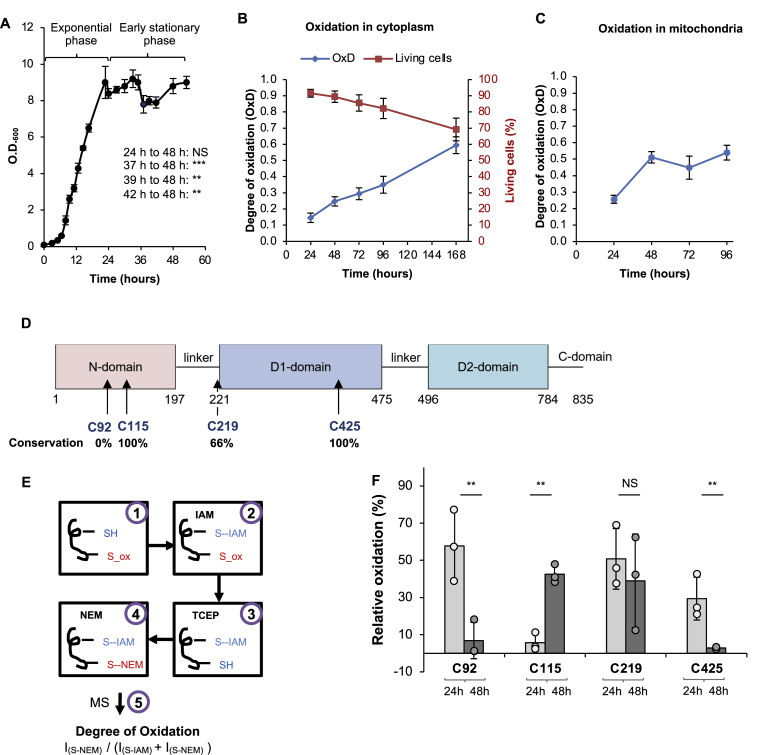
**Elevated cellular oxidation and distinct oxidation profile of Cdc48-Cys115 during the early stages of stationary phase in yeast.** Measurement of yeast OD_600_ during chronological aging (53 h) was done in five biological repeats after dilution from an overnight culture (t = 0 h). The onset of the stationary phase was defined at 24 h after exponential growth, followed by a slight decrease and recovery at 48 h. Statistical significance of the difference in OD_600_ between 24, 37, 39 and 42 h relative to 48 h was evaluated by using Student's t-test (NS- p-value >0.05, ∗∗ - p-value <0.01, and ∗∗∗ - p-value <0.005). (B, C) Cells expressing cytosolic (B) and mitochondrial (C) Grx1-roGFP2 measured every 24 h for their normalized degree of oxidation (OxD) (blue diamonds in B and C), and cellular vitality (percent of living cells) (right axis, red squares in B). For cytosolic Grx1-roGFP2, measurements from four biological repeats were averaged, and for mitochondrial Grx1-roGFP2, measurements from six biological repeats were averaged. Oxidation increases during chronological aging, with the most drastic change during healthy growth occurring between 24 and 48 h. (D) Schematic representation of yeast Cdc48's domains and relative placement of cysteine residues throughout. Conservation rate determined based on alignment between *S. cerevisiae*, *M. musculus*, *D. rerio*, *H. sapiens*, *A. thaliana*, and *D. melanogaster* Cdc48 homologs. (E) Schematic representation of differential thiol-trapping methodology using iodoacetamide (IAM) and N-ethylmalemeide (NEM): (1) Proteins are unlabeled, with either reduced (SH) or oxidized (S_ox) cysteine thiols. (2) Treatment with IAM labels reduced thiols to S--IAM. (3) Treatment with TCEP reduces previously oxidized thiols. (4) Treatment with NEM labels newly reduced thiols to S--NEM. (5) Identification of peptides with the labeled cysteines using mass spectrometry, and calculation of the relative cysteine oxidation based on the ratio between the peak intensities. (F) Percentage of oxidized Cdc48 cysteine-containing peptides at both 24 h (gray) and 48 h (black), derived from the ratio between IAM- and NEM-modification intensities as described in (E). (NS – p-value >0.05, ∗∗ - p-value <0.01, using a Student's t-test. Error bars reflect standard deviation between three biological repeats.)

To characterize these early stages of the stationary phase, we monitored growth, survival, and the redox status of *S. cerevisiae* BY4741 cells in a synthetic medium supplemented with casamino acids. Based on measured O.D._600_ values, we defined the exponential phase (up to ∼22 h), onset of the stationary phase (24 h), and early stationary phase (48 h) (Fig. 1A). Under these growth conditions, we observed a marginal but significant decrease in cellular O.D._600_ between 24 and 48 h of growth, followed by a return to levels similar to those observed at the onset of the stationary phase. Together, this suggested shifted cellular growth and potentially metabolic activity between 24 and 48 h. Importantly, this transition to the stationary phase was not triggered by an inhibition of cellular respiration, as demonstrated by the comparative oxygen consumption rates of cells at 24 h and 48 h of growth (Fig. S1A). Both the 24 h- and 48 h-aged cells exhibited comparable rates of oxygen consumption, with a marginal increase at 48 h growth. Thus, we suggest that these growth conditions allow us to decouple early oxidation and starvation, which is associated with increased oxidation due to fermentation.

To define the redox status of the cells at different stages of stationary growth, we used the *in vivo* encoded Grx1-roGFP sensor to quantify the increase in oxidation levels within the cytosol (Fig. 1B) and mitochondria (Fig. 1C), as previously described [71]. Grx1-roGFP is a ratiometric sensor which monitors changes in intracellular degree of oxidation (OxD), reflected by the glutathione redox potential [72,73]. As expected, cellular oxidation increased over seven days of chronological aging coupled with a decrease in cellular survival. Importantly, cellular oxidation nearly doubled after the onset of the stationary phase (between 24 h and 48 h), increasing from 15 % to 25 % in the cytosol and from 27 % to 50 % in the mitochondria (Fig. 1B and C). Despite this significant oxidative shift, cell death remained minimal (<∼2 %, Fig. 1B, red squares). Interestingly, after 48 h growth, the mitochondrial matrix exhibits a relative stabilization in the oxidation state while the cytosolic oxidation shows an almost linear increase. This might suggest decoupling of these redox events in cytosol and mitochondria after 48 h growth. Thus, the early stationary phase until 48 h is characterized by mild oscillations in growth, almost no cell death (Fig. 1B, red squares), and increased cellular oxidation (Fig. 1B, blue circles). Unfortunately, due to decreased overall expression of the mitochondrial probe at later time points (possibly due to mitochondrial damage accumulated during chronological aging), we were unable to reliably measure mitochondrial oxidation specifically at later time points.

**Fig. 2:**
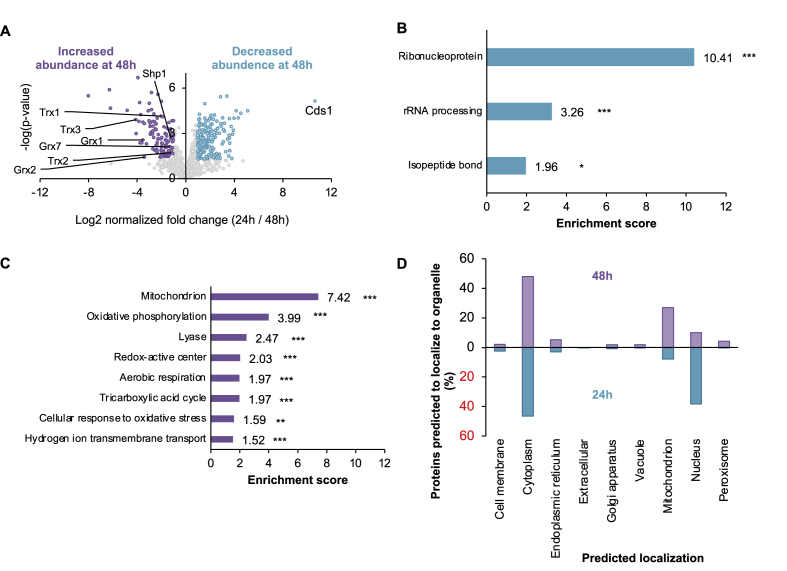
**Cdc48's interactome undergoes major rearrangement during the early stage of stationary phase in yeast.** (A) Volcano plot of the Cdc48 interactome differences between cells grown to 24 h and 48 h. Proteins significantly increased are labeled in purple (left) and while those significantly decreased at 48 h are labeled in cyan (right). Definition of a significant change is based on log2 fold change >1, and FDR<0.05. Non-significant (NS) proteins are labeled in gray. (B, C) Functional enrichment analysis of significantly decreased (B) and increased (C) proteins at 48 h, was conducted by using the DAVID webserver [89]. Significance of the enrichment was marked by stars as following: ∗ - p-value <0.05, ∗∗ - p-value <0.01, ∗∗∗ - p-value <0.005. (D) Localization analysis of proteins significantly enriched in Cdc48's interactome at 24 h (cyan, negative values) or 48 h (purple, positive values) was annotated according to Yeast Genome Database [90] by using the DeepLoc server [91].

Following a redox proteomics analysis of aging yeast cells which defined Cdc48 as a potential age-dependent redox-sensitive protein [14], we mapped redox changes in all Cdc48 cysteines during the early stages of the stationary phase. Yeast Cdc48 has four cysteine residues at positions 92, 115, 219, and 425, of which only two (Cys115 and Cys425) are conserved among unicellular and multicellular eukaryotes, including the yeast and mammalian homologs [74] (Fig. 1D). Notably, Cys115 and Cys425 are located in different functional domains of the Cdc48 protein: Cys115 is positioned at the N-terminal domain, involved in binding of Ubx-type cofactors [62], and Cys425 at the conserved ATPase domain of Cdc48 (Fig. 1D). Previous studies suggested that Cys115 might be redox sensitive as its oxidation increased from 29 % to 55 % during the first two days of chronological aging [14]. To quantify the levels of *in vivo* thiol oxidation of this and all other cysteines during the 24–48 h transition, we conducted a differential thiol-trapping analysis using two alkylation reagents that differ in size (iodoacetamide (IAM) and N-ethylmaleimide (NEM)), followed by mass spectrometry (Fig. 1E and F). After trypsin digestion and peptide identification, we quantified differences in the peak intensities between Cdc48 cysteine-containing peptides harboring either NEM- or IAM-cysteine modifications (Fig. S2A and B). Notably, Cys115 alone showed a major shift in oxidation status from being reduced (5.8 ± 4.7 % oxidation) at 24 h to oxidized (42.5 ± 4.9 %) at 48 h; these results indicated that almost half of Cys115 thiols were reversibly oxidized during the first day of stationary phase (Fig. 1F). In contrast, Cys92, which is also located in the N-terminal domain (Fig. 2A–D) became significantly reduced (from 57.8 ± 19 % to 6.9 ± 9.8 %) during the 24–48 h transition (Fig. 1F). This distinct redox pattern may have functional and structural origins, as Cys115 is less buried than Cys92 (Fig. S2C) and is exclusively located within the UBX binding site of Cdc48. Similarly, Cys425, which is located in the D1-ATPase domain of Cdc48, became increasingly reduced (29.4 ± 11.5 % to 2.9 ± 0.6 %) while the redox state of Cys219 remained unchanged. Structural analysis of these two cysteines in the human VCP (hVCP) structure (5FTN, Fig. S2D) suggested that the distance between the thiols to ATP might be reflected in the oxidation pattern of the nearby cysteine residues. Since Cys219 (Cys209 in hVCP) is located ∼5–9 Å from ATP, its oxidation might alter ATP binding and hydrolysis, while the redox status of Cys425 (Cys415 in hVCP) might be less influential on ATPase activity due to its localization in another cavity and farther away from ATP. The redox proteomic analysis revealed the differential redox pattern of all Cdc48 cysteines in the context of age-dependent, reversible redox modification, which demonstrated that Cys115 harbors distinct features from those of other Cdc48 cysteine residues. This further suggests that Cys115 serves as a molecular switch of Cdc48's activity during intrinsic changes in the cellular redox status.

### 3.2 Alterations in yeast Cdc48's interactome during early stationary phase

To understand the implications of the altered oxidation in the Cdc48 interactome, we identified and quantified changes in the abundance of Cdc48-interacting proteins at 24 h and 48 h growth by comparative proteomic analysis. Specifically, Cdc48 was pulled down with its interacting partners using a C-terminal FLAG tag through co-immunoprecipitation after 24 and 48 h cell growth. Subsequent digestion and LC-MS/MS analysis of these proteins identified 858 proteins among at least three biological repeats (three for 24 h and four for 48 h) (Table S1), whose relative intensities were calculated using label-free quantification (LFQ) via MaxQuant [75]. The pairwise *t*-test analysis revealed that ∼32 % (276 out of 857 co-immunoprecipitated proteins) exhibited significantly altered relative abundance during the 24–48 h transition (using FDR <0.05 and more than 2-fold change (Fig. 2A–Table S1). Notably, Cdc48's interactome rearrangement involves both interactors with increased (115, purple in Fig. 2A) and decreased (161, blue in Fig. 2A) abundance at 48 h, 95 % of which are unique to Cdc48's interactome. Importantly, the observed vast temporal differences in Cdc48's interactome do not reflect proteome changes, which were found to be modest (Fig. S3A, Table S1, Table S2). Specifically, among 1682 proteins identified, after the onset of stationary phase (from 24 to 48 h growth), only ∼3.1 % proteins displayed significantly elevated (50 proteins, ∼2.9 %) or declined levels (5 proteins, ∼0.3 %).

Our interactome analysis identified six known Cdc48 co-factors: Npl4, Ufd1, Shp1/Ubx1, and Ubx2, Ubx3 and Ubx4 (Table S1). While Npl4, Ufd1, Ubx3 and Ubx4 did not show major differences in Cdc48 binding during the 24 h–48 h transition, Shp1/Ubx1 was significantly increased in the 48 h interactome (Fig. 2A). In contrast, Ubx2 binding was slightly decreased (by 1.5-fold, p-value 0.015) at the 24 h growth time point. In addition, some of the identified interactors might be Cdc48 substrates as well as direct or indirect binding partners, which might mediate Cdc48 function and localization.

Functional annotation of the Cdc48 interactors pointed to an increase in proteins involved in redox-based biosynthesis, TCA cycle, mitochondrial functions, and redox homeostasis at 48 h, alongside a decrease in ribosomal functions (Fig. 2B and C). While Cdc48 levels were similar at the 24–48 h transition, its association with cytosolic and mitochondrial antioxidant proteins such as glutaredoxins (Grx1, Grx2, Grx7), thioredoxins (Trx1, Trx2, Trx3), and peroxiredoxin (Tsa1) was increased at 48 h. Association with various mitochondrial proteins was similarly elevated, as seen in the analysis of the proteins with the most significantly increased abundance (Table S1). However, the abundance of the nuclear thioredoxin peroxiredoxin Dot5 declined in the 48 h Cdc48 interactome. Moreover, none of these proteins was correspondingly increased in the whole cell proteome between 24 h and 48 h.

Localization analysis of the significantly changed proteins suggested a shifted interaction profile between 24 h and 48 h, with Cdc48 associating with less nuclear proteins during the second day of the stationary phase (38 % at 24 h to 10 % at 48 h), alongside an increase in association with the mitochondrial proteins (8 % at 24 h to 26 % at 48 h) (Fig. 2D). The potential shift in Cdc48's localization was further supported by annotation enrichment analysis, indicating a shift from association with ribonucleoproteins and ribosomal proteins toward interactions with mitochondrial and respiration processes (Fig. S3B). It is important to note that such predictions are limited by the assumption that proteins do not play multiple roles within the cell, such that at this time we can only speculate as to direct changes in Cdc48 localization. Unfortunately, attempts to directly validate changes in Cdc48 localization were unsuccessful, due to negative effects of the GFP-tag on Cdc48 function in normal and stress conditions. Similar attempts to pull down Cdc48 from specific subcellular compartments (e.g., the ER, nucleus, etc.) suffered from inconsistently low protein levels, poor fractionation, and changes in the nature of the organellar fraction itself at different experimental timepoints. Notably, attempts to isolate microsomes resulted in pellets of different consistencies and colors between 24, 48, and 72 h of cellular growth (results not shown). While we cannot draw conclusions from these experiments, we may hypothesize that their failure is in fact part of a dynamic reshaping that takes place in the cell during the stationary phase, as seen in Cdc48's overall proteomic interactions during aging.

Taken together, these findings suggest that Cdc48 plays a previously unknown role in the age-dependent cellular redox homeostasis.

### 3.3 The thiol group of Cdc48-C115 is crucial for the oxidative stress response

**Fig. 3:**
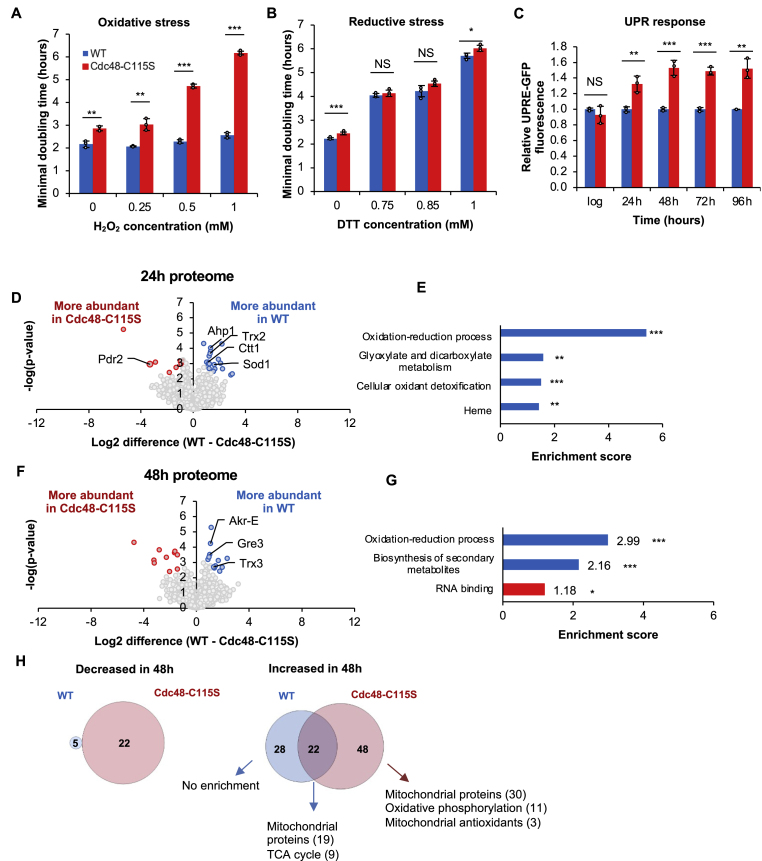
**Cdc48 regulates the oxidative stress response in a Cys115-dependent manner.** (A) Growth of cells under increasing levels of hydrogen peroxide (H_2_O_2_) in either the WT or Cdc48-C115S strains. Cdc48-C115S has a severe growth inhibition under oxidative stress as measured by the strains' minimal doubling time (MDT) compared to no significant change in the WT's growth. (B) Cdc48-C115S growth is not affected by reducing stress through increasing DTT concentrations. (C) The unfolded protein response (UPR) increases in Cdc48-C115S relative to the WT upon entrance to the stationary phase (24 h) and further during the extended stationary phase (up to 96h). Error bars for (A–C) represent the standard deviations of means from three biological repeats. Significance was determined by a Student's t-test, with p-value cutoffs as follows: NS, not significant – p-value >0.05, ∗ - p-value <0.05, ∗∗ - p-value <0.01, ∗∗∗ - p-value <0.005. Dashed lines denote inset for zoom. (D, F) Volcano plot of a global proteomic comparison of WT and Cdc48-C115S cells after 24 h and 48 h growth (D and F, respectively), with several antioxidants and stress-associated proteins changed between the two strains. (E, G) Functional enrichment analysis of proteins significantly more abundant in the WT proteome at 24 h and 48 h (E and G, respectively), showing increased oxidation-reduction functions. (H) Venn diagrams of the overlap between proteins significantly changed in the WT and Cdc48-C115S proteomes between 24 h and 48 h. At 48 h, the Cdc48-C115S variant has a unique enrichment for many mitochondrial proteins.

To assess the potential role of the redox sensitive Cdc48-Cys115 residue on the cellular ability to cope with oxidative stress, we used yeast cells expressing the Cdc48-C115S variant. We previously showed that this mutation decreased degradation of Cdc48 substrates during the log phase, led to a mild upregulation of UPR at 24 h, and decreased longevity [74]. Here, we extended this analysis and found that cells expressing the Cdc48-C115S variant exhibited significantly impaired growth during oxidative stress conditions (Fig. 3A). Notably, the C115S mutation escalated the growth defect under oxidative stress induced by increasing levels of hydrogen peroxide (H_2_O_2_), which severely delayed Cdc48-C115S growth, contrasting the almost null effect under normal conditions (Fig. 3A, Cdc48-C115S in red vs. WT in blue). Importantly, this effect was specifically linked to oxidative stress; there was no additional growth impairment during reducing stress (treatment with dithiothreitol (DTT)) (Fig. 3B), starvation (0.5 % glucose instead of 2 %) (Fig. S4A), or heat shock (growth at 37 °C, where Cdc48-C115S grew slightly faster than WT) (Fig. S4B). Moreover, this effect was not limited to externally induced oxidation stress. As we have previously shown, stationary phase cells are heterogeneous in their intrinsic redox status. Using flow cytometry, we isolated intrinsically oxidized and reduced WT and CDC48-C115S cells, as determined by changes in the Grx1-roGFP probe after reaching the stationary phase under normal growth conditions [70]. These cells were then resuspended in a synthetic medium and their growth rates (doubling times) were compared. The mutation in Cys115 reduced the growth of intrinsically oxidized cells compared to WT, while not affecting reduced and the predominantly reduced unsorted populations (Fig. S4C). Furthermore, using the GFP-based reporter for UPR element (UPRe) expression as previously described [70], we observed no difference in UPRe activation in the WT and Cdc48-C115S cells during the log phase. However, the C115S mutation significantly increased UPR activation during the stationary phase and prolonged chronological aging, particularly after 48 h (Fig. 3C).

To further explore the effect of the Cys115 thiol removal on the sensitivity to oxidative stress, we examined the mitochondria, which are some of the main factories of cellular oxidants. Mitochondria were imaged using roGFP fused to the matrix-targeting mitochondrial signal (Su9). WT and Cdc48-C115S cells revealed similar mitochondrial morphology under normal conditions. However, 1 mM H_2_O_2_ exposure led to punctate, fragmented mitochondria in nearly all Cdc48-C115S cells (Fig. S4D, marked by arrows) while most WT cells maintained unaltered mitochondria. In line with these results, at 48 h growth, Cdc48-C115S-expressing cells presented a disrupted and punctate mitochondrial network (Fig. S4D), indicating a potential impact on the fitness decline in Cdc48-C115S cells during oxidative stress and chronological aging.

### 3.4 The Cdc48-C115S mutation alters temporal proteomic profiles of mitochondrial and redox homoeostasis proteins

To further challenge the working hypothesis that the Cdc48-C115S substitution may introduce global changes in cells, we conducted a proteomic analysis comparing the Cdc48-C115S variant to WT cells after 24 h and 48 h growth (Fig. 3D–H, Tables S3–S4). The removal of the Cys115 thiol led to a mild impact on the protein profile, altering the abundance of 26 out of 1731 proteins and 22 out of 1716 proteins at 24 h and 48 h growth, respectively. Notably, a majority of the Cdc48-C115S-driven decreased proteins are antioxidants (e.g., Sod1, Ahp1, Trx1, Ctt1) and mitochondrial proteins (Fig. 3D and E).

Moreover, the Cdc48-C115S substitution led to a significant increase in Pdr1, a transcriptional factor involved in hypoxia and stress response [26]. After 48 h growth, the Cdc48-C115S mutation led to a decrease of the mitochondrial thioredoxin (Trx3), aldolase reductase Gre3, and NADPH-dependent a-keto-amide reductase, Akr-E, all of which are involved in the cellular defense against oxidative stress (Fig. 3F and G). This correlates with the observed sensitivity of the Cdc48-C115S cells to oxidative stress, peroxide-triggered change in mitochondrial morphology, and upregulated UPR that already takes place at early stages of the stationary phase.

Comparing the temporal changes in the proteome profiles of WT and Cdc48-C115S strains showed that of 1823 identified proteins, the levels of 22 were elevated in both strains, compared to 48 whose levels were exclusively elevated in the mutant strain after 48 h of growth (Fig. 3H–S5A, Tables S1 and S5). Remarkably, among these, 30 were mitochondrial proteins, 11 of which are involved in oxidative phosphorylation (Table S5). This aligns with the observed peroxide-induced dysmorphology of mitochondria in the Cdc48-C115S strain.

Considering the increased sensitivity of Cdc48-C115S to oxidative stress conditions and the previously described changes in association with antioxidants, these results implicate Cdc48 as a significant player in redox homeostasis. Furthermore, these findings emphasize the specific significance of the redox-sensitive C115 residue in mediating this role.

### 3.5 The Cdc48-C115S mutation alters the age-dependent Cdc48 interactome

**Fig. 4:**
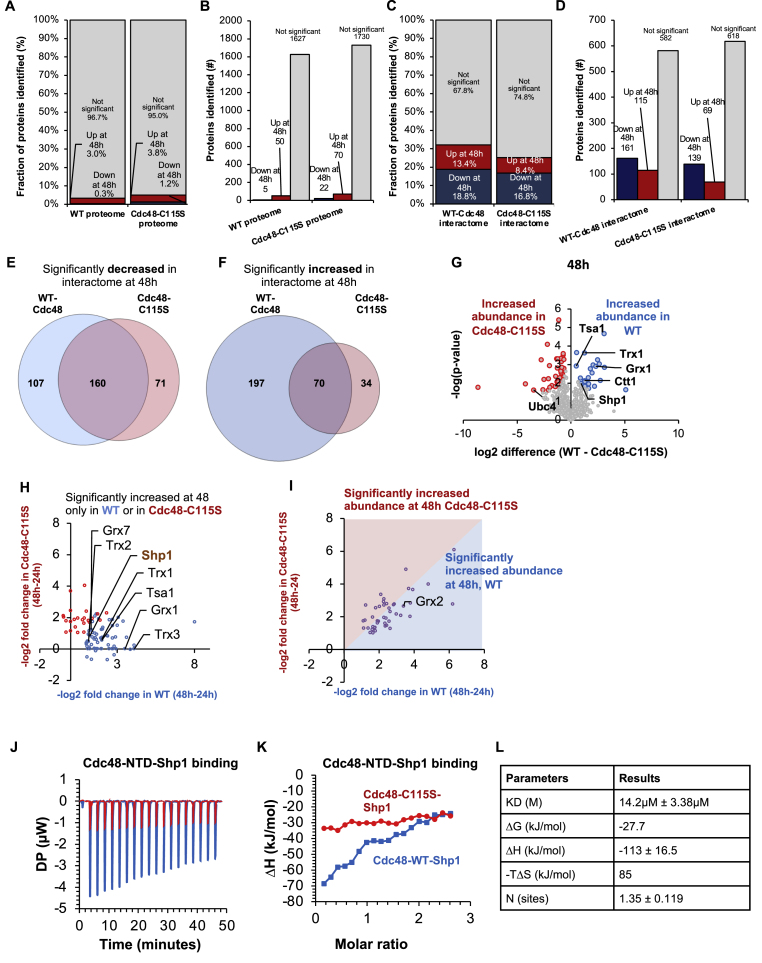
**The Cdc48-C115S mutation alters the age-dependent Cdc48 interactome**. (A–B) Fraction (A) and number (B) of significantly changed proteins in either the WT and Cdc48-C115S proteomes, between 24 h and 48 h. (C–D) Rate (C) and number (D) of significantly changed proteins in the Cdc48 interactome of either WT or Cdc48-C115S cells. For (A–D), proteins that are significantly down at 48 h are in dark blue, significantly up at 48 h are in red, and those with no significant change are in gray. (E–F) Venn diagram of significantly changed proteins in WT (blue) and Cdc48-C115S (red) interactome at 24 h (E) and 48 h (F). (G) Volcano plot of direct comparison of the Cdc48-WT and Cdc48-C115S interactomes at 48 h. Many antioxidants have notably increased abundance in the WT interactome, as well as Cdc48's canonical cofactor Shp1/Ubx1. (H) Correlation plot of proteins that are significantly more abundant in only either the WT (blue) or Cdc48-C115S (red) interactomes, and not found or not significantly altered in the other strain. (I) Correlation plot of fold change differences in the WT and Cdc48-C115S interactomes. Proteins that were significantly increased in both the WT and Cdc48-C115S (pink background) at 48 h are shown. Proteins that showed a relatively higher difference in the WT Cdc48 interactome as compared with Cdc48-C115S at 48 h are in the blue area, while proteins that are more abundant in the Cdc48-C115S are in the pink area. The plot represents data from Table S9. (J–K) Isothermal titration calorimetry (ITC) measurements of the binding affinity of WT (blue) and C115S (red) Cdc48-NTD proteins with Shp1/Ubx1. The binding affinity was determined by titrating 220 μM of Shp1/Uxb1 into 16 μM of either WT or -C115S Cdc48-NTD. The C115S mutation abolished binding with Shp1/Ubx1, resulting in significantly reduced heat change produced from the binding events (J) and no change in corresponding molar ratio shown by the titration curve (K). (L) Fitted parameters of the ITC measurements, corresponding to J and K.

Intrigued by these results, we decided to examine the age-dependent interactome of Cdc48 in the context of the temporal Cys115 oxidation. For this purpose, we conducted a comparative interactome analysis of WT and Cdc48-C115S cells cultivated for 24 and 48 h under the same conditions described in Fig. 1. The Cdc48-C115S interactome included 826 identified proteins, 208 of which were significantly changed at either 24 or 48 h (Fig. S5B, Table S6). Here, we observed a similar shift in the predicted protein localization between 24 h and 48 h as in the WT (Fig. S5C). In contrast to the moderate changes in the whole cell temporal proteomic analysis (Fig. 4A and B), an opposite trend was observed for Cdc48's interactome in the context of Cys115 mutation. Mutation in C115S slightly decreased the number of interactors in the Cdc48 interactome, resulting in a lower fraction of interactors at 48 h, a time period that is associated with Cys115 oxidation. Specifically, 231 (13.4 %) proteins showed stronger interaction with WT at 48 h compared to 24 h, but only 103 (8.4 %) proteins interacted with Cdc48-C115S (Fig. 4C, D, E, F).

The expedited aging of mutated cells could further extend to mitochondrial aging. Supporting this notion, mutated C115S led to decreased binding of ten mitochondrial proteins already at 24 h, and another four at 48 h. Those proteins included key redox homeostasis regulators, such as Sod1, Trx2, Ctt1 and Ahp1 in the 24 h interactome, and Trx3 in the 48 h interactome (Fig. 4G–S6A, Tables S3 and S4).

Since Cysteine, but not Serine at the 115 position undergoes oxidation during the 24 h–48 h transition, we expected redox-associated changes between Cdc48 WT and Cdc48-C115S interactomes to mainly emerge at 48 h, which was indeed found to be the case (Fig. 4G–I; corresponding data for 24 h in Fig. S6B–C). The observed differences included proteins that interacted only with Cdc48 WT but not Cdc48C115S (Fig. 4H, blue), as well as proteins that underwent larger changes in the WT interactome than in Cdc48-C115S (Fig. 4G, under the line Table S7). Intriguingly, several of the unique interactors whose levels were elevated in WT at 48 h are crucial for the oxidative stress response and oxidation-associated mitochondrial functions (e.g., Tsa1, Trx2, Trx3, Grx1) (Table S9) or mediate PQC and proteostasis (Fig. S5D).

Taken together, these findings indicate a priming role of Cdc48 interactome rearrangement under accelerated oxidative stress response, and specifically following Cys115 oxidation during the shift from early to late stationary phase.

Among canonical cofactors, Shp1/Ubx1 was the only one significantly affected by the Cdc48-C115S mutation. Shp1/Ubx1's abundance was elevated in the Cdc48-C115S interactome at 48 h, albeit less pronouncedly than in WT (fold change 1.8 with a p-value of 0.015 in Cdc48-C115S, but 2.4 with a p-value of 0.002 in WT) (Fig. 4H).

To validate the role of the Cdc48-C115S mutation toward binding with Shp1/Ubx1, we measured the binding affinity of the purified Shp1/Ubx1 and the Cdc48-NTD domain (Cdc48-NTD) using Isothermal Titration Calorimetry (ITC) (Fig. 4J–L). Titration of 220 μM of Shp1 to 16 μM Cdc48-NTD-WT resulted in a calculated *K*_d_ of 14.2 μM ± 3.38 μM (Fig. 4H, I, blue curve). Unlike WT, mutated Cdc48-C115S showed no binding to Shp1/Ubx1 under the same conditions (Fig. 4H, I red curve). This can be explained by the central location of Cys115 near the binding interface between the Cdc48-NTD and Shp1-Ubx domains (Fig. S7A).

### 3.6 The Cdc48-C115S mutation affects the temporal post-translational modification profile of Cdc48-interacting proteins

**Fig. 5:**
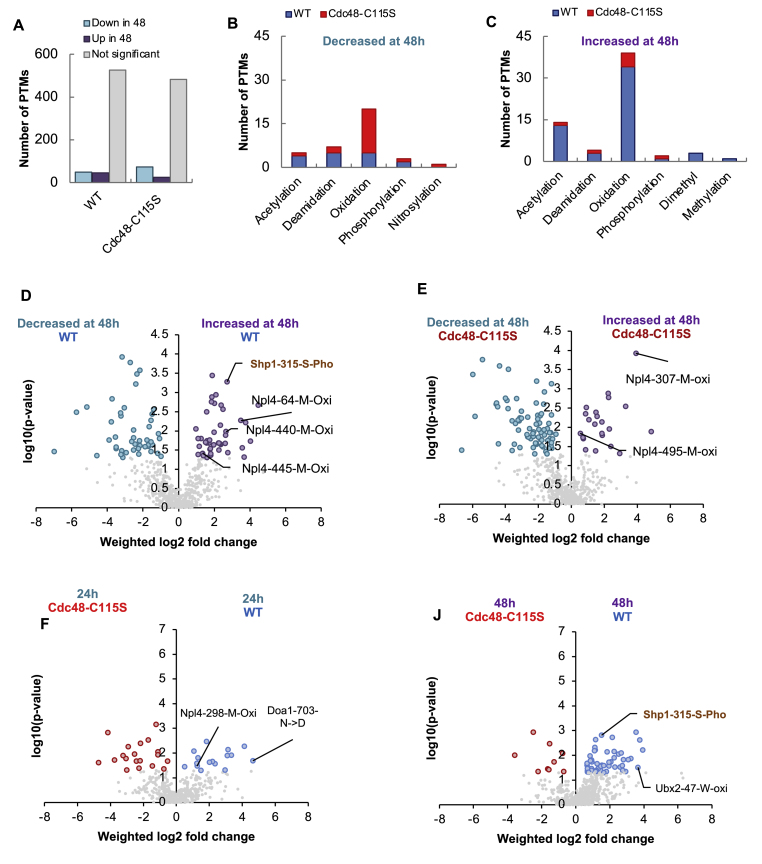
**Cdc48's interaction modificome changes during the extended stationary phase.** (A) Number of identified modifications in the WT and Cdc48-C115S interactome. A majority (525 in WT and 483 in Cdc48-C115S) of the modifications did not significantly change between the 24 h and 48 h interactomes (gray), while abundance of 49 and 73 PTMs (WT and Cdc48-C115S, respectively) decreased at 48 h (cyan) and 45 and 24 PTMs ((WT and Cdc48-C115S, respectively) increased at 48 h (purple). (B, C) Dissection of the PTM types among significantly decreased (B) or increased (C) at 48 h. The major change is in oxidative modifications including mainly oxidation of methionine, as well as oxidation of Tryptophan, Histidine, and tri-oxidation of cysteine (Table S9). (D–J) Volcano plots of different modifications identified on either WT Cdc48's (D) or Cdc48-C115S’ (E) interactomes between 24 h and 48 h. Direct comparison of the WT and Cdc48-C115S interactomes at 24 h (F) and 48 h (J) shows that mainly WT interactome at 48 h has an increased level of modified proteins. The highly increased phosphorylation on Shp1/Ubx1 at Serine315 is labeled, and found more dominant in the WT interactome at 48 h.

Seeking to characterize post-translational modifications (PTMs) of the Cdc48-interacting proteins during aging (24 h and 48 h) in the WT and Cdc48-C115S we analyzed the same interactome data using the PROMISE workflow [76] (Fig. 5A–D, Table S10). We identified a wide range of PTMs in Cdc48's interacting proteins, including mono- and di-oxidation, phosphorylation, nitrosylation, acetylation, ubiquitination and deamidation.

715 PTMs were identified in the WT Cdc48 interactome between 24 h and 48 h, compared to 641 in the Cdc48-C115S interactome, such that ∼15–16 % of the identified modifications were significantly changed during the 24–48 h timeframe (Fig. 5A–Table S10). This analysis highlighted significantly increased oxidative modifications of Cdc48's interactors (mainly on Methionine) at 48 h as compared with 24 h (Fig. 5B and C). Among these, Cdc48-C115S appeared to associate far less tightly with oxidatively-modified proteins than WT Cdc48, specifically after 48 h (Fig. 5) (Table S10). This correlated with the Cys115 oxidation, in line with the general increase in intrinsic cellular oxidation. Crucially, several of these modifications were on major Cdc48 cofactors such as Shp1/Ubx1, Npl4, and Ubx2 (Table S10, Volcano plots, Fig. 5D–J). These modifications were all either undetected or insignificant at 24 h. At 48 h, comparing the WT and Cdc48-C115S strains revealed stronger WT interaction with modified proteins, including Ubx2, Shp1/Ubx1, Npl4, ribosomal proteins, and others (Fig. 5D–J, Table S10). Importantly, phosphorylated Shp1/Ubx1 interacts more strongly with WT Cdc48 a day after the onset of the stationary phase (48 h) (Fig. 5D), suggesting that this interaction depends on the Cys115 modification (Fig. 5J).

Interestingly, only three ubiquitinated proteins were identified: two ribosomal proteins, Rpl43A, Rps31, and the Cdc48 co-factor, Ufd1. None of these modifications were found to be enriched chronologically or under Cdc48-Cys115 mutation. This may indicate that non-specific, ubiquitinated client proteins interacting with the Cdc48 co-factors, and directly targeted to degradation constitute a very modest fraction of the Cdc48 interactome described in this study.

Together with data from Cdc48's interaction with antioxidant proteins, this analysis raises important questions regarding a potential role of Cdc48 in the triage and turnover of oxidized proteins, possibly through Cys115 oxidation.

### 3.7 Shp1-S315 phosphorylation cooperates with Cdc48-Cys115 oxidation

Intrigued by the potential effect of Cys115 on temporal binding between Cdc48 and Shp1/Ubx1 *in vivo* and *in vitro* (Fig. 2, Fig. 5) as well as on the interaction with the phosphorylated Shp1/Ubx1 mainly at 48 h in WT cells, we pursued potential cooperativity of Cys115 and Shp1-Ser315 phosphorylation.

**Fig. 6:**
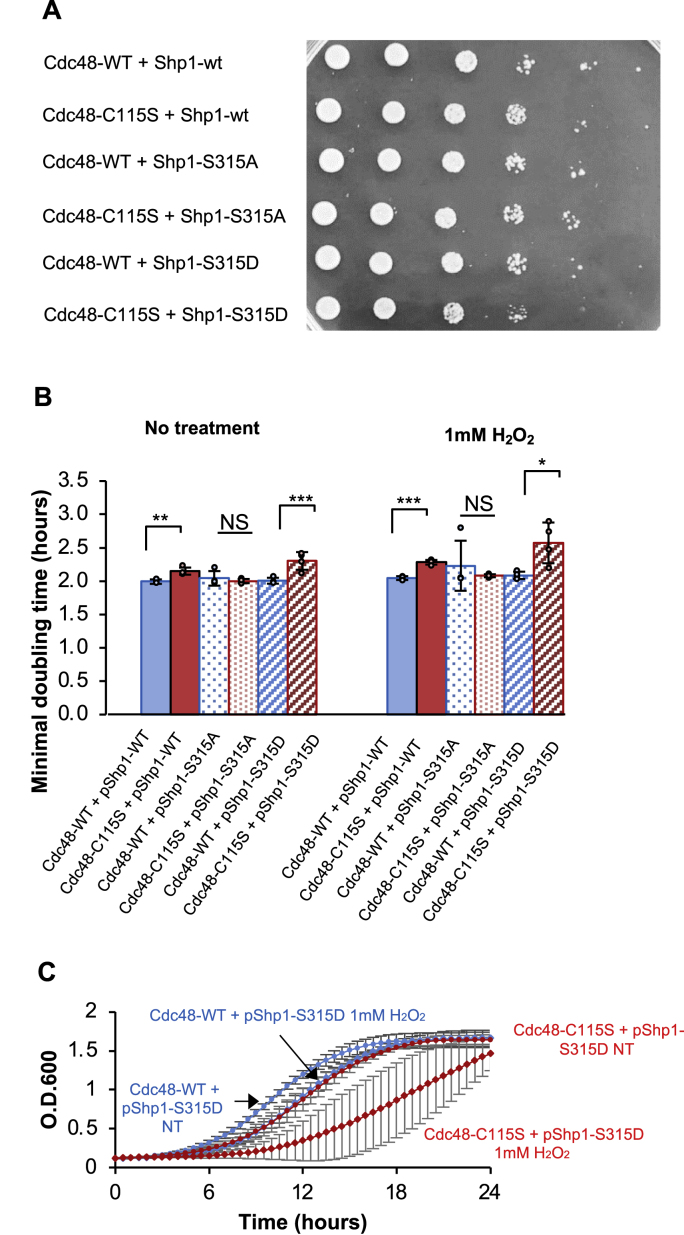
**Shp1-Ser315 cooperates with Cdc48-Cys115 in mediating the oxidative stress response.** (A) Serial dilutions of WT and Cdc48-C115S strains complemented with pShp1-WT/S315A/S315D spotted onto selective plates and recovered for 2 days at 30 °C. (B) Minimal doubling time WT and Cdc48-C115S strains (similar to A) grown in absence or presence of 1 mM hydrogen peroxide (H_2_O_2_). Cdc48-C115S with pShp1-S315D has an exacerbated growth inhibition under oxidative stress as shown in the growth curve in (C). The minimal doubling time was calculated from growth curves in (C) and Fig. S6B. Error bars represent the standard deviations of the means from four biological repeats, with a Student's t-test conducted for significance. P-value cutoffs: NS (non-significant) – p-value >0.05, ∗ - p-value <0.05, ∗∗ - p-value <0.01, ∗∗∗ - p-value <0.005. Dashed lines denote inset for zoom.

To test this, we produced CDC48-WT/Δ*shp1* and CDC48-C115S/Δ*shp1* strains by knocking out the *SHP1* gene*.* To assess its potential effect on Shp1-Ser315, we mimicked the phosphorylation modification by substituting Serine with a negatively charged Aspartic acid (Shp1-S315D). We then tested the fitness of the designed strains by expressing either wild type Shp1, Shp1-S315D, or Shp1-S315A proteins from a plasmid (pShp1, pShp1-S315D and pShp1-S315A) under a native promoter (Fig. 6). In line with a previous study [77], we found that mutated Ser315 in Shp1/Ubx1 alone had little impact on cellular growth under normal or mild oxidative treatment (Fig. 6A and B, blue bars). However, Cdc48-C115S demonstrated clear negative cooperativity with the phosphorylated-like Shp1 (pShp1-S315D), resulting in significantly impaired growth with pShp1-S315D during normal and oxidative stress conditions (Fig. 6A, B (striped bars), Fig. 6C–S7A, B). Interestingly, pShp1-S315A complementation in Cdc48-C115S had either modest or null improvement of cellular fitness. Together, these results suggested that in cells lacking the redox-sensitive Cdc48-Cys115 protein, Shp1-Ser315 phosphorylation disrupts growth and the cellular redox response.

## 4 Discussion

In natural ecosystems, the majority of prokaryotic and eukaryotic cells exist in a stationary phase, defined by reduced transcriptional, translational, and metabolic activity. Entry into this stationary phase allows cells to cope with environmental stresses, including heat and oxidation, by rewiring the proteostasis network to maintain properly folded proteins and to degrade damaged ones. To explore the mechanisms enabling this quiescence, we focused on an early time window within the chronological lifespan, prior to the extended aging, which is characterized by global protein oxidation, decreased intracellular NADPH and GSH levels [14,15], accelerated acidification, nutrient depletion and protein degradation [15]. Cultivating cells in standard medium supplemented with casamino acids allowed us to zoom into the very initial stages of the stationary phase, differentiating between its onset (24 h growth) and early stage (48 h). During this transition, cellular oxidation increased by nearly 2-fold (OxD is 15 % vs 25 % in cytosol and 27 % vs 50 % in mitochondria), with almost no impact on cell survival. Here, we investigated the role of Cdc48 and its site-specific oxidation in the UBX-binding domain during the increasing intrinsic oxidation of the first days of the stationary phase.

### 4.1 Temporal dynamics of the Cdc48 interactome during the postmitotic stage initiation

The transition from onset of the stationary stage to its early stage led to mild changes in the proteome, primarily in mitochondrial proteins such as the cytochrome C complex, and TCA cycle proteins. The proteomic profile did not reflect oxidative stress conditions, as redox homeostasis proteins, heat shock, and ribosomal proteins were not altered. In contrast, Cdc48's interaction profile underwent substantial differences from 24 h to 48 h, emphasizing changes in the Cdc48 environment and client proteins and suggesting a dynamic adaptation of the Cdc48-dependent degradation process during the early stages of the stationary phase.

Predictably, mitochondrial functions were globally elevated between the early and extended stationary phases. These changes were accompanied by altered Cdc48's specific functions: one day following the stationary phase onset (48 h), Cdc48 revealed novel mitochondrial associations and fewer nuclear associations (Fig. 1). This likely reflected elevated Cdc48-dependent degradation of mitochondrial proteins under the increased mitochondrial oxidation, as monitored by the Grx1-roGFP sensor. Unfortunately, multiple attempts to monitor Cdc48's subcellular localization during the stationary phase stages were not successful. Specifically, GFP fusion to Cdc48 led to altered Cdc48 abundance and sensitivity to oxidative stress, while organellar fractionation was hampered by challenges in reproducibility and organelle isolation purity. Thus, we can currently only speculate as to direct changes in Cdc48's localization, as characterized by an altered interaction profile.

Our study also identified a previously unknown yet prominent changes in aging-related Cdc48's interactomes, culminating in a tightened association with antioxidants and redox-related proteins, such as the thiol-restoring proteins Trx1-3, Grx1,2 and 7, Pdi1, and the peroxiredoxin Tsa1.

Apart from the enrichment of redox-related proteins in Cdc48's aged interactome, we identified enriched ribosomal and ribonucleo-protein complexes in the early stationary phase. Cdc48 is known to regulate ribosomal-associated degradation [78], a function that may markedly decline in cells entering the stationary phase. Correspondingly, several proteins with tightened Cdc48 association during the extended stationary phase aligned with previous aging-related studies e.g., Phosphoenolpyruvate carboxykinase (Pck1 [[16], [79]]) and mitochondrial proteins, including the Succinate dehydrogenase (Sdh2 [80]) and citrate synthase (Cit1 [81,82]), members of the cytochrome complex. Moreover, PTM analysis of Cdc48's interactors suggested that this timeframe involved increased oxidation of Cdc48-interacting proteins, likely including its client proteins, protein-degradation regulators, such as Npl4, Ubx2, and others. Notably, the majority of these modified proteins did not change in abundance during the early stages of the stationary phase, with only a small fraction (less than 10 %) showing alterations.

Timewise, our data suggests that Shp1/Ubx1 interaction with Cdc48 increases after the onset of the stationary phase, potentially playing a role in its ability to cope with the escalating cellular oxidants. Notably, yeast Shp1/Ubx1 is homologous to four different human co-factors: p37, p47, UBXN2A, and UBXN11, all sharing the SHP domain that mediates interaction with VCP/p97 through its N-terminal domain [83]. A recent study showed that p37, rather than p47, mediates the cytosol and nucleus shuttling of VCP/p97 [84], suggesting differential functions of various Shp1/Ubx1 homologs. Examining the role in protein turnover of p37 and other human Shp1/Ubx1 homologs during senescence and aging, particularly under oxidative stress can assist in unraveling the temporal regulation of subcellular localization of Cdc48 and its special function.

### 4.2 Cys115 of Cdc48 is a potential redox modulator

Advances in redox proteomics have enabled mapping of potential redox-regulatory proteins across different species, suggesting that a vast majority of cysteine-containing regions may play regulatory functions in various processes, including protein homeostasis and stress response [13,36]. However, only a few of those redox-regulated cysteines have been experimentally explored. One such example is Cdc48. Already a decade ago, Cys115 of Cdc48 was found to undergo age-dependent oxidation during the early stages of chronological aging in DBY746 *S. cerevisiae* cells [14]. Cys115 was identified as an “early oxidized” residue, whose oxidation was doubled during the first day of the stationary phase (when ∼90 % of other protein thiols remained unmodified). Moreover, the early oxidation of Cys115 occurred a day before the general collapse in protein thiol oxidation during normal and caloric stress conditions, and was sustained under different growth conditions. Here, we found that this age-dependent oxidation is unique among other Cdc48 cysteines and confirmed this finding in another *S. cerevisiae* strain (BY4741) and slightly different growth medium.

Our study further demonstrated that a single point mutation in Cdc48 sufficed to trigger a global effect in terms of the cells’ ability to cope with oxidative stress conditions (both externally induced and endogenously produced). In this context, the Cys115 thiol is selectively pivotal for redox-related stress (e.g., aging and oxidative stress), and not for other proteotoxic stresses, such as heat shock and reductive stress, defining its potential role as a thiol-redox switch. Using a comparative interactome analysis, we identified a subset of Cdc48 interactors that uniquely bind the WT and Cdc48-C115S proteins, together identifying these interactions as potentially dependent on Cys115 and its redox status. This subset of proteins included antioxidants and the Cdc48 cofactor Shp1/Ubx1, which associated predominantly with the oxidized form of Cdc48 (WT, 48 h).

While it seems unlikely that Cdc48's interactions with antioxidants are direct, their changing abundance relative to Cdc48 strongly suggests that Cdc48 exerts Cys115-dependent regulation of cellular oxidation at large. Whereas oxidative stress was already tightly linked with PQC [29,30,37], Cdc48 has not until now been directly implicated in preventing or mitigating the effects of increased cellular oxidation. Our current finding shows that its involvement is at least partly Cys115-dependent, and structural examination suggests that Cys115 oxidation may facilitate its role as a redox switch, as it is located in the flexible loop within the Cdc48-Ubx binding interface [85]. Unfortunately, we were unable to examine this biochemically, as our attempts to purify the Cdc48 variant which can partially mimic Cys115 oxidation (Cdc48-C115D) resulted in an unstable and insoluble protein.

Our analysis of Cdc48-Cys115's oxidative status could not identify irreversibly oxidized modifications (e.g., S-carbonylation or sulfonylation), distinguish between reversible subtypes of oxidized cysteine (disulfide bond or sulfenylation), or identify redox-related cysteine-specific modifications, such as palmitoylation, S-nitrosylation, and others. Those are of particular importance, as palmitoylation in human VCP and S-nitrosylation in plants have previously been identified on the homologous cysteine residue by specialized proteomic analysis [[86], [87], [88]].

Importantly, numerous aging-related changes in the Cdc48 interactome are Cys115-independent. Those remain a fascinating avenue for further identifying the general functional role of Cdc48 in the stationary stage. Although these modifications have not yet been validated in our redox-regulating context, it would be interesting to further explore their roles.

Furthermore, our results implicate Cdc48's cofactor Shp1/Ubx1, showing a crosstalk between post-translational modifications of Cdc48-Cys115 and Shp1-Ser315 in cellular survival under oxidative stress conditions. We show that Shp1/Ubx1 not only increases its association with Cdc48 during the stationary phase (48 h), but also elevates Ser315 phosphorylation, which further emerges during proteotoxic conditions induced by TORC1 inhibition [77]. Co-expression of Cdc48-C115S with the phosphorylation-mimicking variant Shp1-Ubx1 S315D decreases cellular survival, suggesting a potential incompatibility of phosphorylated Shp1/Ubx1 interaction with the reduced form of Cdc48-Cys115.

**Fig. 7:**
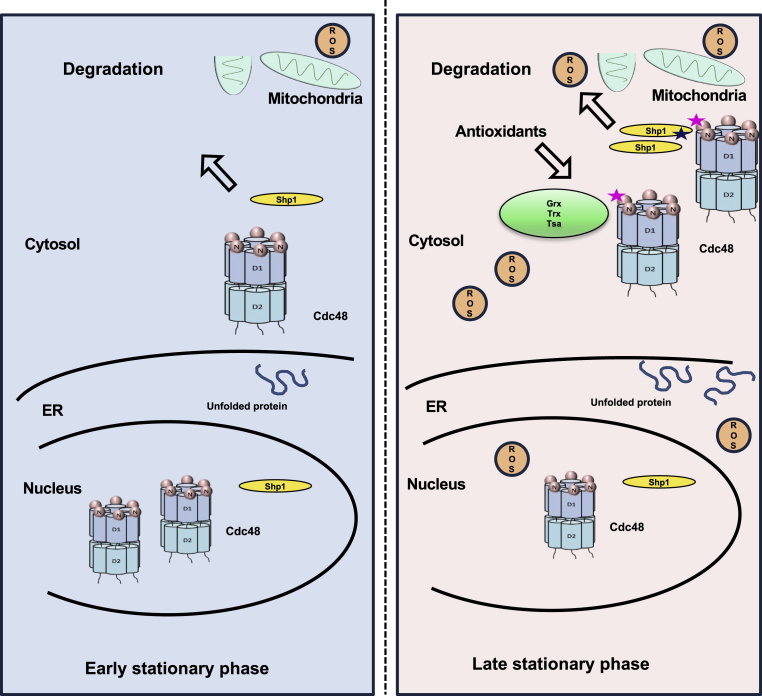
**Schematic model for Cdc48 behavior in the shift to oxidative conditions.** At the early stationary phase (blue), Cdc48 prominently interacts with nuclear and ribosome-associated proteins. Upon oxidation of Cys115 during the later stationary phase (corresponding with an increase in cellular ROS), Cdc48 increases association with mitochondrial proteins, antioxidants, and cofactor Shp1.

Based on this multilevel analysis, we propose a model by which Cdc48 responds to a changing cellular redox environment through its Cys115 residue (Fig. 7). Under non-stress conditions, Cdc48 may be found in both the nucleus and cytosol, with moderate association with mitochondria. As the cell undergoes oxidation during chronological aging and Cdc48 is recruited to a wider range of subcellular compartments, it associates more closely with Shp1/Ubx1, which undergoes phosphorylation of Ser315. Mutation of Cys115 appears to reduce cellular ability to regulate many oxidative stress-related functions. This raises many new research directions regarding Cdc48/VCP/p97's role in cellular aging and age-related pathologies, alongside broader implications for PQC members in regulating distinct cellular processes.

## CRediT authorship contribution statement

**Meytal Radzinski:** Writing – review & editing, Writing – original draft, Visualization, Validation, Methodology, Investigation, Formal analysis, Data curation, Conceptualization. **Tal Oppenheim:** Writing – review & editing, Writing – original draft, Validation, Investigation, Formal analysis, Data curation. **Ohad Yogev:** Investigation, Formal analysis, Conceptualization. **Adi Levy:** Investigation, Data curation. **Melamed-Book Naomi:** Investigation. **Assaf Kacen:** Software, Methodology, Formal analysis, Data curation. **Yifat Merbl:** Supervision. **Tommer Ravid:** Supervision, Resources, Methodology, Investigation. **Dana Reichmann:** Writing – review & editing, Writing – original draft, Visualization, Supervision, Software, Resources, Methodology, Investigation, Funding acquisition, Formal analysis, Conceptualization.

## Author disclosure statement

The authors state that no competing financial interests exist.

## Declaration of competing interest

The authors declare the following financial interests/personal relationships which may be considered as potential competing interests: Dana Reichmann reports financial support was provided by 10.13039/501100003483The Hebrew University of Jerusalem. If there are other authors, they declare that they have no known competing financial interests or personal relationships that could have appeared to influence the work reported in this paper.

## Data Availability

Data will be made available on request.
