# Supplementary material for: Cdc48 plays a crucial role in redox homeostasis through dynamic reshaping of its interactome during early stationary phase
Source: Redox Biol. 2025 May 1;84:103651. doi: 10.1016/j.redox.2025.103651 (PMC12137173; doi:10.1016/j.redox.2025.103651)
Supplement: Multimedia component 1 [file mmc1.pdf]

Supplementary material

Supplementary tables (attached separately).

Supplementary table 1. Comparative proteomic analysis of the WT Cdc48 interactome at 24h and 48h growth.

Supplementary table 2. Comparative proteomics of the WT strain at 24h and 48h growth.

Supplementary table 3. Comparative proteomics of WT and Cdc48-C115S strains at 24h growth.

Supplementary table 4. Comparative proteomics of WT and Cdc48-C115S strains at 48h growth.

Supplementary table 5. Comparative proteomics of Cdc48-C115S at 24h and 48h growth.

Supplementary table 6. Comparative proteomic analysis of Cdc48-C115S interactome at 24h and 48h growth.

Supplementary table 7. Comparative interactome analysis of WT and Cdc48-C115S strains at 24h growth.

Supplementary table 8. Comparative interactome analysis of WT and Cdc48-C115S strains at 48h growth.

Supplementary table 9. Comparison of significantly altered binding proteins of WT and Cdc48-C115S.

Supplementary table 10. The temporal post-translational modifications (PTMs) analysis of the WT and Cdc48-C115S binding proteins.

Supplementary figures:

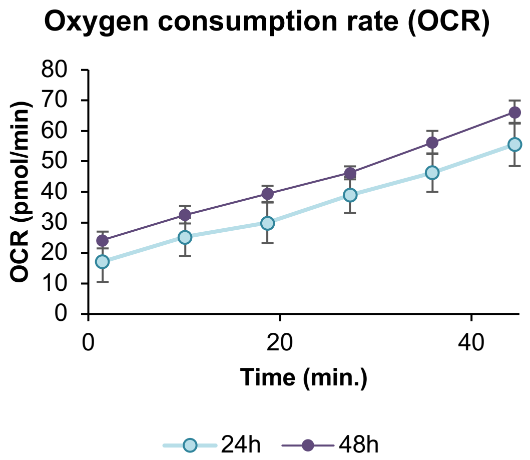

Supplementary Figure 1, related to Figure 1A. Yeast cells exhibit similar oxygen consumption after entering the stationary phase (24 h) and one day after (48 h). Oxygen consumption rates (OCR) of cells grown for 24 h (purple) and 48 h (blue) were measured by Seahorse XFp Flux Analyzer (Seahorse Bioscience) as described in[1]. Error bars reflect standard deviation of three biological repeats.

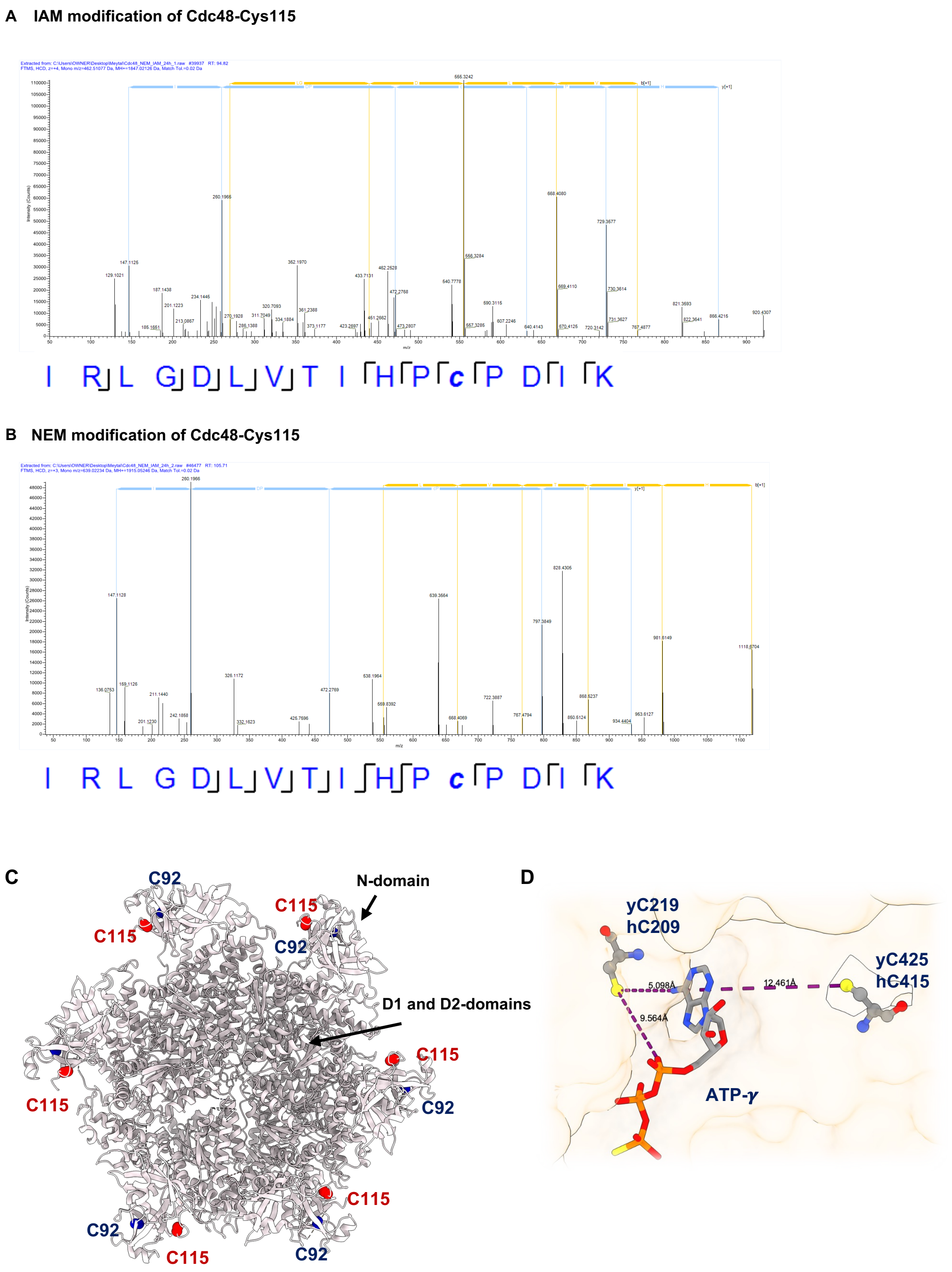

Supplementary Figure 2, related to Figure 1D-F. Mapping the temporal oxidation profile of Cdc48 cysteines using differential labeling. (A) Representative annotated MS/MS spectrum of an IAM-modified (A) and NEM-modified (B) tryptic peptide containing Cdc48-Cys115, related to either reduced or oxidized cysteine, respectively, randomly

chosen among the relevant peptides from three biological repeats as analyzed by Proteome Discoverer (Thermo Fisher Scientific). The modified cysteine is indicated by a lowercase letter. (C) Localization of Cys92 (blue) and Cys115 (red) in the N-terminal domain of yeast Cdc48 hexamer (PDB ID: 6OPC). (D) Cys219 is positioned closer to the ATP pocket than Cys425. The structure of the human homolog, hVCP, with the ATP-γ ligand (PDB ID: 5FTN) was used for the visualization. The yeast Cys219 (yC219) corresponds to human Cys209 (hC209), and yeast Cys425 (yC425) corresponds to human Cys415 (hC415).

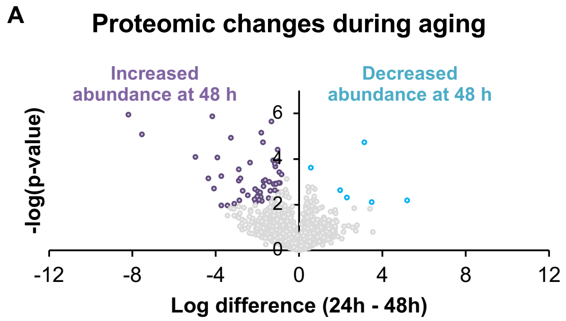

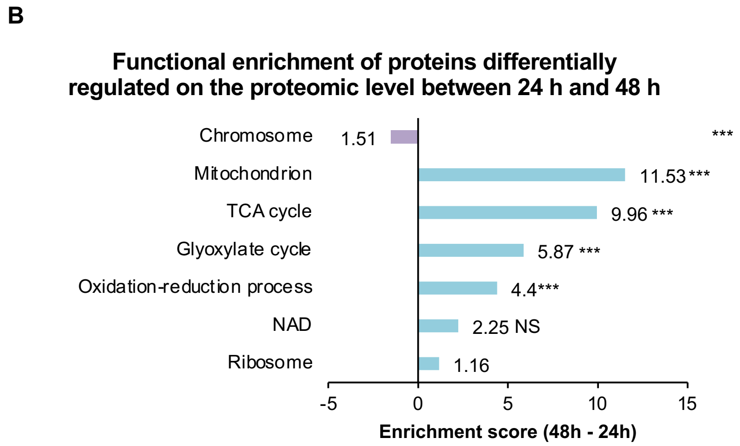

Supplementary Figure 3, related to Figure 2. Comparative proteomics of cells grown to 24 h and 48 h reveals mild changes in the proteome level, with an increase in mitochondrial and redox-related proteins. (A) Volcano plot of global proteomic differences between cells grown to 24 h and 48 h, according to an FDR of 0.05. (B) Functional enrichment analysis of significantly changed proteins in the aging proteome between 24 h and 48 h. Functions enriched at 24 h are in purple (left) and those enriched at 48 h are in blue (right). Significance is based on the DAVID enrichment p-value of the respective function. NS – p-value > 0.05, *** - p-value < 0.005

Supplementary 4

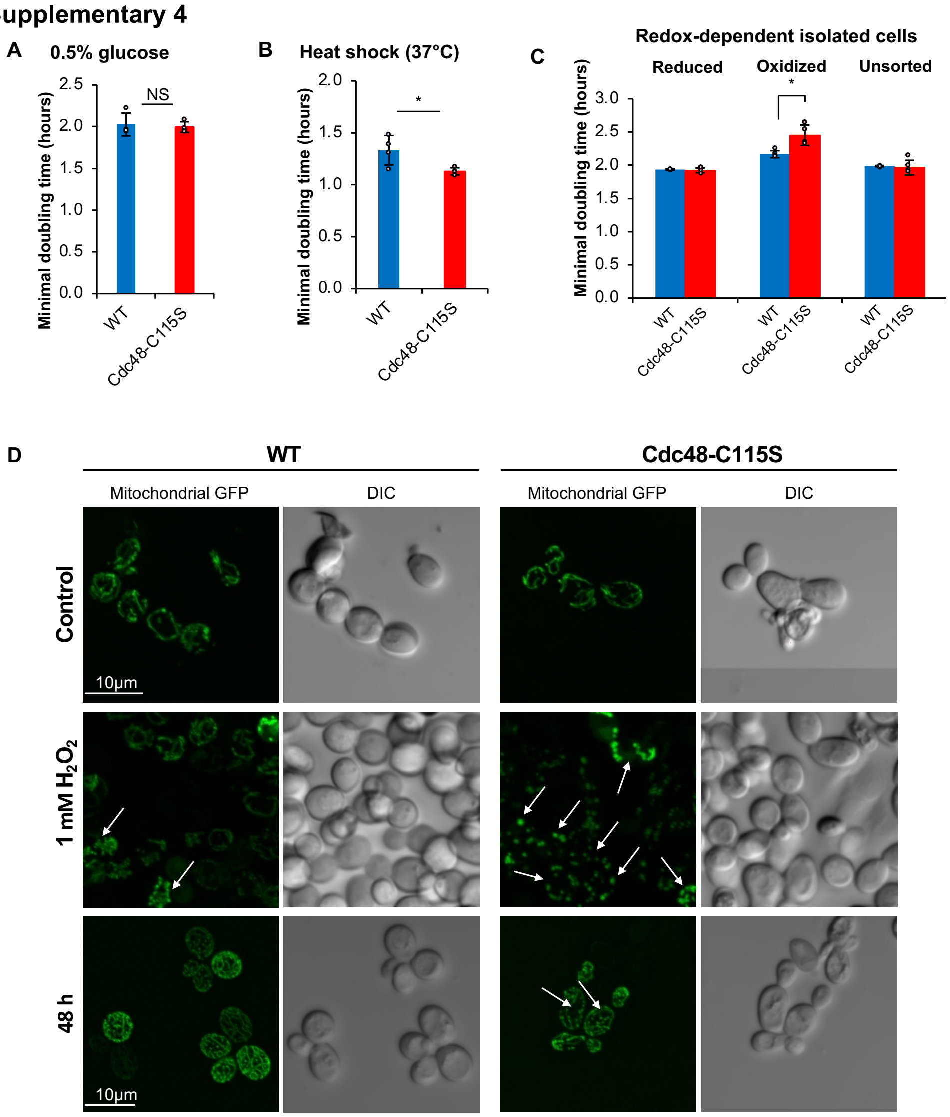

Supplementary figure 4, related to Figure 3. The effect of Cys115S mutation is specific to oxidative stress conditions.

(A-B) Cdc48-C115S did not affect yeast growth either under lower glucose (A) nor heat stress (37ºC) conditions (B). (C) Growth of subpopulations of cells isolated based on their native redox status as measured by the Grx1-roGFP2 probe (oxidized (405/488 ratio > 2) and reduced (405/488 < 1.5)), as well as an unsorted (mixed) population. Cdc48-C115S growth is impaired under endogenously oxidized conditions while there is no difference for reduced cells. Significance for (A-C) was determined by a Student's t-test between four biological repeats, with p-value cutoffs as follows: NS – p-value > 0.05, * - p-value < 0.05, ** - p-value < 0.01, *** - p-value < 0.005. Dashed lines denote inset for zoom. (D) Mitochondrial morphology is altered in Cdc48-C115S under mild oxidative stress. Treatment for 1 hour with 1 mM H_2O_2 triggers widespread mitochondrial fragmentation in the Cdc48-C115S variant, in contrast with only a few such cases in the WT. At 48h, mitochondria were comprised of a dense network in the WT, and disturbed in Cdc48-C115S.

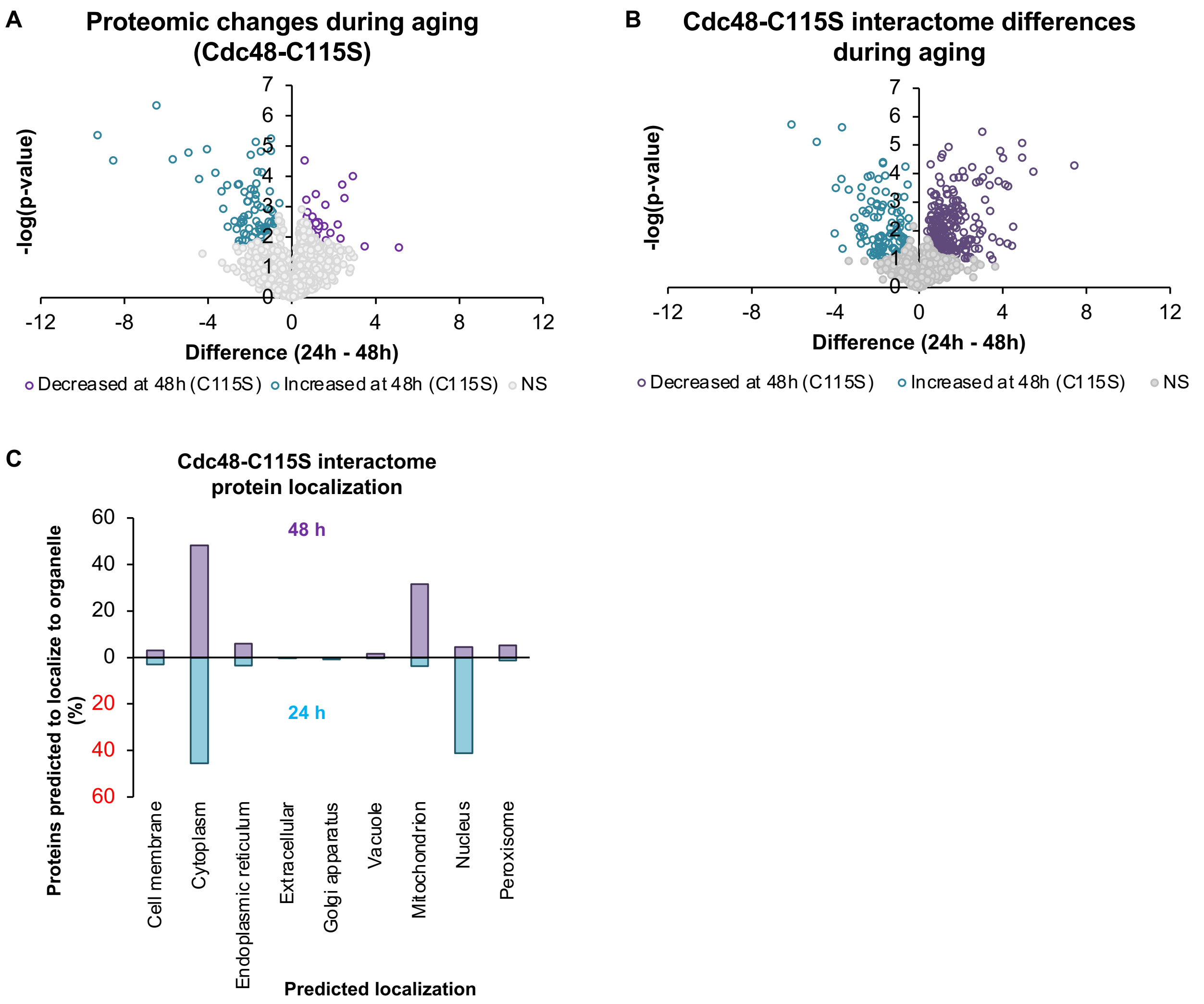

Supplementary Figure 5, related to Figure 4. The Cdc48-C115S mutation alters the age-dependent Cdc48 interactome (A-B) Proteomic (A) and Cdc48 interactome (B) changes during aging in Cdc48-C115S, with proteins significantly increased at 24h in blue (right) and those significantly increased at 48h in red (left), corresponding to changes in the WT in Fig. S1B and Fig. 2A, respectively. (C) Changes in predicted localization of proteins significantly enriched in Cdc48-C115S's interactome at 24 h (blue, negative values) or 48 h (red, positive values).

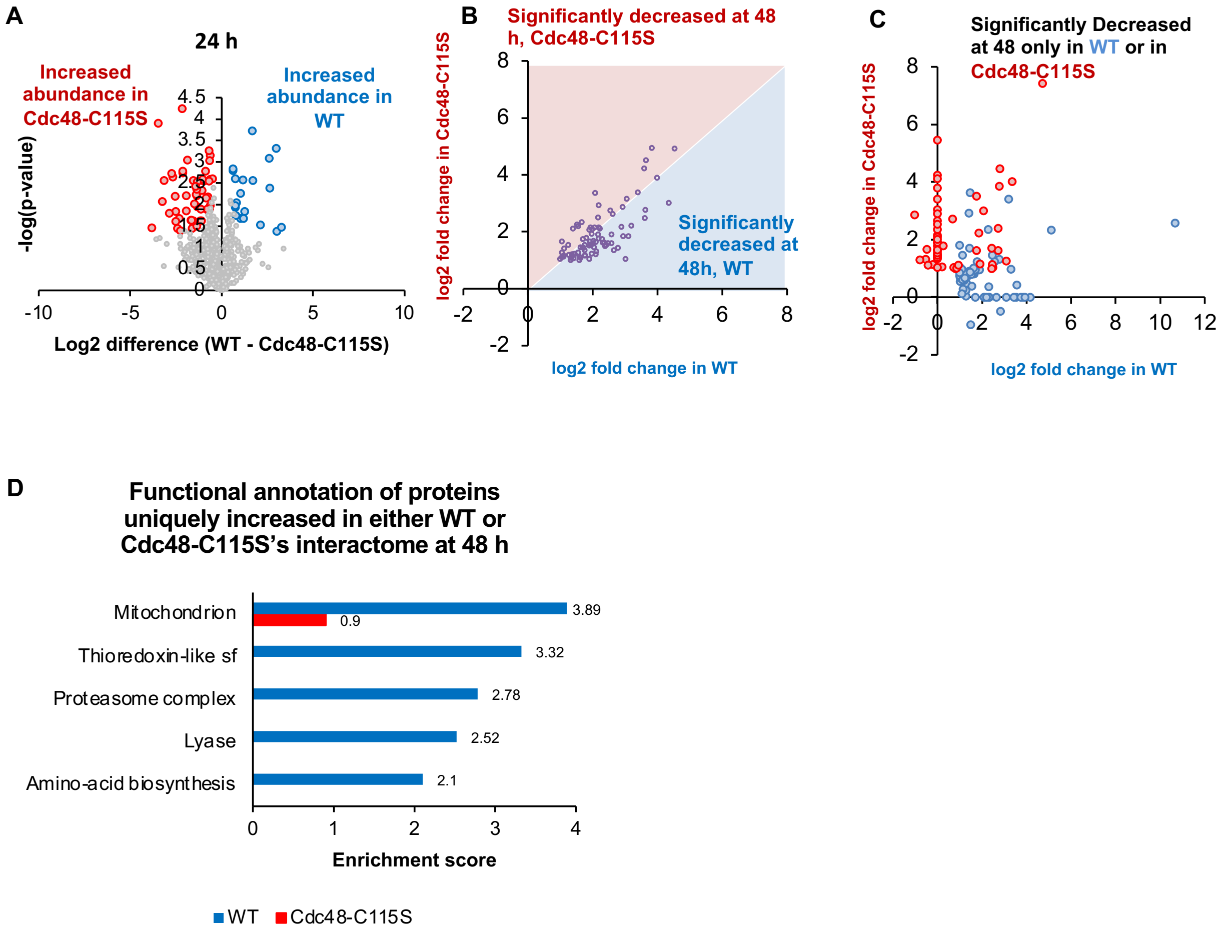

Supplementary figure 6, related to Figure 4. The Cdc48-C115S mutation alters the age-dependent Cdc48 interactome.

(A) Volcano plot of direct comparison of the Cdc48-WT and Cdc48-C115S interactomes at 24 h. (B) Correlation plot of protein differences in the WT and Cdc48-C115S interactomes that are significantly decreased in both the WT and Cdc48-C115S at 48 h. The color code is as in Fig. 4I. (C) Correlation plot of proteins with that are significantly less abundant in only either the WT (blue) or Cdc48-C115S (red) interactomes at 48 h. (D) Functional enrichment analysis of uniquely significantly increased proteins in the Cdc48 interactome at 48 h for both the WT (blue) and Cdc48-C115S (red).

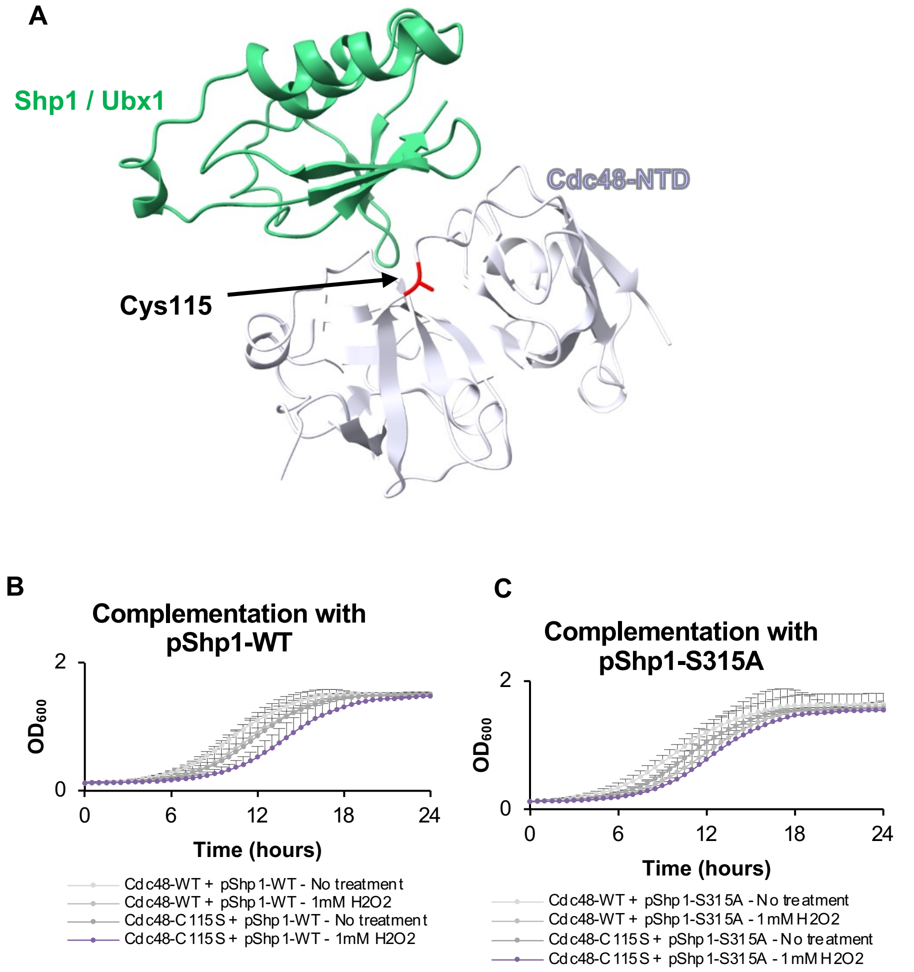

Supplementary Figure 7, related to Figure 6. Cys115 affects the Shp1/Ubx1-Cdc48 interaction.
(A) Structure of Shp1/Ubx1 (green) in complex with Cdc48's N-terminal domain (grey), highlighting Cys115 (red) facing the interaction interface (based on PDB ID: 6OPC).
(B-D) Growth curves of WT and Cdc48-C115S complemented with either pShp1-WT (B) or pShp1-S315A (C) in four repeats. Data are represented as mean ± standard deviation in four biological repeats.

Reference:

1. Radzinski, M. *et al.* Temporal profiling of redox-dependent heterogeneity in single cells. *eLife* **7**, e37623 (2018).
